# Different Niemann-Pick C1 Genotypes Generate Protein Phenotypes that Vary in their Intracellular Processing, Trafficking and Localization

**DOI:** 10.1038/s41598-019-41707-y

**Published:** 2019-03-28

**Authors:** Hadeel Shammas, Eva-Maria Kuech, Sandra Rizk, Anibh M. Das, Hassan Y. Naim

**Affiliations:** 1 Department of Physiological Chemistry, University of Veterinary Medicine Hannover, 30559 Hannover, Germany; 2 Clinic for Paediatric Kidney-, Liver-, and Metabolic Diseases, Hannover Medical School, 30625 Hannover, Germany; 3 Department of Natural Sciences, Lebanese American University, Beirut, 1102-2801 Lebanon

## Abstract

Niemann-Pick Type C (NP-C) is an inherited neurovisceral lysosomal storage disease characterized by a defect in the trafficking of endocytosed cholesterol. In 95% of patients the gene encoding NPC1 is affected. The correlation of the genetic background in NP-C with the clinical phenotype such as, severity and onset of liver dysfunction, ataxia, dystonia and vertical gaze palsy, has not been elucidated at the molecular level. We have designed strategies to investigate the effect of different mutations in the NPC1 gene at the protein and cellular levels. The NPC1 mutants were expressed in mammalian cells and their structural features, maturation pathways and subcellular localization elucidated. Interestingly, three classes of NPC1 mutants could be identified and further characterized. The first group comprised mutants in which the NPC1 protein revealed virtually similar structural features to the wild type species. It was trafficked to the lysosomes and colocalized with the lysosomal protein marker Lamp2. The second class of NPC1 mutants was only partially trafficked to the lysosomes, but predominantly localized to the endoplasmic reticulum (ER). In the third group with the most severe phenotype, NPC1 mutants were entirely retained in the ER, colocalizing with the ER-protein marker calnexin. In conclusion, this study relates NPC1 mutations to the trafficking behavior of the NPC1 mutants along the secretory pathway. The findings are essential for a comprehensive understanding of the pathogenesis of NP-C and propose a mutation-based personalized therapeutical approach.

## Introduction

Niemann-Pick Type C disease (NP-C) is caused by a defect in the trafficking of endocytosed cholesterol with sequestration of unesterified cholesterol in lysosomes and late endosomes^1^. Other lipids also accumulate with variation between different tissues: cholesterol and sphingomyelin storage is most abundant in peripheral tissues, while glycosphingolipid (GSL) buildup is most significant in the central nervous system^2^.

NP-C disease can affect the viscera (liver, spleen, and sometimes lung) and central nervous system with most common clinical symptoms (over 70% of cases) being clumsiness, learning difficulties, ataxia, dysphagia, dysarthria and vertical gaze palsy^3^. NP-C is caused by an autosomal recessive mutation in one or both of the *NPC1* and *NPC2* genes, with NPC1 gene mutations being present in 95% of cases^4^. The clinical spectrum of NP-C ranges from neonatal potentially fatal liver failure to an adult-onset neurodegenerative disease^1^. The incidence of severe NPC1 is approximately 1/92,104 live births and estimated at 1/19,000-1/36,000 for the late-onset NPC1 phenotype^5^.

Clinical diagnosis is difficult due to the diversity of clinical symptoms and the wide range in age at clinical onset. Staining of cultured fibroblasts from skin biopsies with filipin, a fluorescent sterol binding compound, which detects lipid accumulation in the lysosomes is frequently performed, supplemented by genetic testing^6,7^. In recent years, identification of several sensitive plasma biomarkers elevated in NP-C (e.g. cholestane-3β,5α,6β-triol, lysosphingomyelin isoforms and bile acid metabolites), has been added to the spectrum of diagnostic procedures, but did not offer a gold standard test for NP-C^8^. In fact, high oxysterol levels can be unspecifically found in neonatal cholestasis, thus cholestasis might be a pitfall^9^.

Increasing evidence has demonstrated that NPC1 and NPC2 proteins sense and coordinate the transport of cholesterol out of the late endosome/lysosome system^1^. Recent resolution of the crystal structure of human NPC1 and computational modeling suggested that a cavity in the sterol-sensing domain is large enough to accommodate one cholesterol molecule^10^. NPC1 is essentially involved in Ebola virus (EBOV) infection being its intracellular receptor. A 4.4 Å structure of full-length human NPC1 resolved the mode of binding of EBOV through domain C^11^. NPC1 may also play a role in the retrograde transport of various lipids and proteins from late endosomes to the trans-Golgi network^12^.

The NPC1 protein, localized in the late endosomes and lysosomes, is associated with the lysosomal membrane via 13 transmembrane segments^13^. It is a highly glycosylated protein that harbors 14 potential asparagine residues for N-linked glycosylation type of the sequence Asn-X-Ser/Thr^14^. The biosynthesis and trafficking pathways of NPC1 resemble those of many other membrane and secretory glycoproteins and implicate co-translational glycosylation and correct folding in the endoplasmic reticulum (ER), transport to the Golgi apparatus and processing to a mature complex glycosylated protein^15^ and finally targeting to the lysosomes via the dileucine motif in the C terminal of NPC1 protein^14^.

More than 400 disease-causing mutations covering virtually the entire protein sequence of NPC1 have been described. The pathophysiological processes underlying different clinical features (juvenile vs late onset, visceral vs neurological symptoms) have not yet been elucidated. Since NPC1 is directly involved in lysosomal cholesterol trafficking, it can be hypothesized that an imbalanced or impaired trafficking due to a mutation confers global alterations on the cellular membrane composition^16^. Particularly, lipid rafts, a special group of membrane microdomains, could be affected. Lipid rafts are ordered liquid domains rich in sphingolipids and cholesterol which segregate from less-ordered liquid domains composed of mainly unsaturated phospholipids^17^. They can be extracted using nonionic detergents and thus are also called detergent-resistant membranes (DRMs). Regulatory roles have been assigned to lipid rafts in signaling and trafficking pathways^18–20^.

At present, the iminosugar N-butyldeoxynojirimycin (NB-DNJ) (Miglustat) is the only disease modifying option which reversibly inhibits glucosylceramide synthase, thus reducing storage phenomena^21,22^. Miglustat has several drawbacks since it only temporarily stabilizes various clinically relevant manifestations of NP-C^23^, and it is associated with gastrointestinal side effects reducing the quality of life^24–26^. Nevertheless, the severity of clinical conditions at the beginning of treatment justifies the use of this therapeutic option^27^.

In the present study, we analyzed the impact of a group of common mutations in infantile, juvenile and late-onset NP-C on structural features, intracellular localization and trafficking with the ultimate goal of defining their pathogenicity at a biochemical level. These mutations are located in different regions of the NPC1 protein encoding the transmembrane domains (TMD), luminal domains, cysteine-rich loop and sterol sensing domain. In a second step, we tried to correlate the biochemical phenotype with the clinical phenotype. Differences in the protein trafficking, intracellular localization and membrane association of NPC1 mutants would be compatible with variations in the pathomechanisms associated with the onset of NP-C.

Better understanding of the pathophysiological processes and their association with the clinical symptoms in NP-C may lead to the discovery of novel, more specific and sensitive biomarkers for diagnosing and monitoring the disease.

## Materials and Methods

### Reagents

Dulbecco’s Modified Eagle’s Medium (DMEM), fetal calf serum (FCS), methionine-free DMEM, streptomycin, penicillin, protease inhibitors, trypsin, trypsin-EDTA, DEAE-dextran, protein A-sepharose, and triton X-100 were purchased from Sigma-Aldrich. Tissue culture dishes were purchased from Sarstedt (Nuembrecht, Germany). [^35^S]-methionine was obtained from PerkinElmer (Waltham, Massachusetts). Acrylamide, Tris, TEMED, SDS, dithiothreitol (DTT), paraformaldehyde, polyvinyl difluoride (PVDF) membrane, ProLong Gold Antifade mountant with DAPI by Invitrogen, and sucrose were purchased from Carl Roth GmbH (Karlsruhe, Germany). Lubrol WX was obtained from MP Biomedicals (Eschwege, Germany). Endo-β-N-acetylglucosaminidase H (endo H) was acquired from Roche Diagnostics (Mannheim, Germany). TRIzol reagent (Life Technologies, California, USA), restriction enzymes, Isis proofreading DNA polymerase, molecular weight standards for SDS-PAGE, and SuperSignal™ West Femto maximum sensitivity western blot chemiluminescence substrate were purchased from Thermo Fisher (Schwerte, Germany).

### Immunochemical reagents

For immunoprecipitation and immunoblotting monoclonal mouse anti-Flag antibody and for immunofluorescence staining both mouse anti-Flag and rabbit anti-Flag antibodies were used (Both Sigma-Aldrich). Co-localization was done using rabbit polyclonal anti-calnexin antibody from Abcam, mouse anti-GM130 for Golgi staining from BD Transduction Laboratories and mouse monoclonal anti-Lamp2 antibody from Abcam. Secondary antibodies were either conjugated to Alexa Fluor dyes (Invitrogen) or horseradish peroxidase (Thermo Fisher). For lipid raft analysis flotillin-2 (B-6) antibody (Santa Cruz) was used.

### Construction of cDNA clones

Total RNA was extracted from Caco-2 cells using TRIzol reagent (Life Technologies), cDNA synthesized using SuperScript™ IV First-Strand Synthesis (Thermo Fisher, Massachusetts, USA) and human NPC1 amplified using primers linked to NotI and XhoI recognition sites:

NPC1_NotI_fw: ACGTGCGGCCGCACCATGACCGCTCGCGGCCTG

NPC1_XhoI_rev: ACGTCTCGAGGAAATTTAGAAGCCGTTCG

NPC1 cDNA was ligated into pCMV-3Tag-3 (Agilent technologies, California, USA). The resulting plasmid NPC1-Flag was used for site-directed mutagenesis PCR to create NPC1 mutations. PCR was performed using Isis polymerase. The NPC1-cDNA amplified primers and the site-directed mutagenesis oligonucleotides are listed in (Table 1). PCR products were treated with DpnI to remove the methylated template and transformed into *E.coli* DH5α. Successful mutagenesis was verified by DNA sequencing.

**Table 1:** Sequences of the forward oligonucleotides used for mutagenesis PCR of the cDNA encoding NPC1 (the reverse oligonucleotides have the reverse complementary sequence) and the localization of the mutations analyzed within the NPC1 protein. TMD, transmembrane domain.

No.	Mutation	Oligonucleotide sequences used for mutagenesis (5′–3′)	Localization of mutations within the NPC1 protein
1	P1007A	CTTTCGGATAACGCTAACCCCAAGTGTG	Cysteine-rich loop
2	D948Y	GGCTGCAACAATTATTCCCTGGTGC	Cysteine-rich loop
3	L1213F	GGATTGTGGTGTTCGCTTTTGCCAAATCTCAGATCTTCCAGA TATTCTACTTCAGG	TMD12
4	V950M	TTTCGACTGGATGAAGCCACAGTCGTC	Cysteine-rich loop
5	M631R	TGTAATTAGCTATGCCATCAGGTTTCTATATATTTCGCTAGC CTTGGGGCACATG	Sterol sensing Domain/TMD 3
6	D874V	CCTACATGGTGGTTTATTTTAAATCCATCAGTCAGTACCTGC ATGCG	Cysteine-rich loop
7	C1168Y	GCATCTCCGTGGAATTCTACAGCCACATAACCAG	Cytosolic loop/TMD11
8	G1162A	GATGAGCTGTGCCATCTCCGTGGAATTCTGCAGCCACATAA CC	TMD11
9	V378A	CACAACCAATCCAGCAGATCTCTGGTCAGCC	Luminal domain 2
10	R404Q	CCTTTCTTCCAGACGGAGCAGCTC	Luminal domain 2
11	H510P	GACTTCTTTGTATACGCCGATTACCCCACGCACTTTCTG	Luminal domain 2
12	Q775P	GACTTTCTTCTGCCGATTACATGTTTCGTGAGTCTCTTGGG	TMD7
13	I1061T	GAAAGCCCGACTTACAGCTAGCAATGTCACCGAAACC	Cysteine-rich loop
14	M1142T	AACATGTTTGGAGTTACGTGGCTCTGGGGCATCAGTTTAA ACGCTGTATCCTTGGTC	TMD10
15	N1156S	GCTGTATCCTTGGTAAGCTTGGTGATGAGCTGTGGC	TMD11
16	G1162V	G1162V GATGAGCTGTGACATCTCCGTGGAATTCTGCAG CCACATAACC	TMD11
17	R1186H	CGCGTGGAGCACGCGGAAGAGGC	Cytosolic
18	L1244P	GGTCTTACTGGGCGCCACTCACGGATTAATATTTCCCCCT GTCTTACTCAG	TMD13

### Cell culture, transient transfection and biosynthetic labeling

COS-1 cells were cultured in DMEM containing 1000 mg/liter glucose supplemented with 10% (v/v) FCS, 100 units/ml penicillin, and 0.1 mg/ml streptomycin. Transient transfection of NPC1-Flag constructs was performed using DEAE dextran as described before^28^, which ensures an overexpression of the NPC1 constructs, minimizing thus a potential effect of endogenous NPC1 on the biochemical phenotype of the mutants. 48 h after transfection, cells were lysed and used for experiments. For biosynthetic labeling 48 h post-transfected cells were starved in methionine-free media for 2 h and labeled with [^35^S]-methionine for 1 h. The plates were washed twice with 1 × PBS and cold medium with 2.5 mM methionine was added. Cells were processed at the indicated time points.

### Cell lysis, immunoprecipitation and deglycosylation

Cell lysates were prepared and immunoprecipitated as described before^29^. Briefly, transfected COS-1 cells were lysed in 25 mM Tris-HCl, pH 8.0, 50 mM NaCl buffer containing 0.5% Na-deoxycholate, 0.5% Triton-X 100 and protease inhibitors. Homogenization was carried out by passing the cell lysate 20 times through a 21G needle. The homogenate was then kept shaking for 4 h at 4 °C. Afterwards the post nuclear supernatant (lysate) was used for further experiments. Immunoprecipitation of Flag-tagged NPC1 using anti-Flag antibodies followed by treatment with endo H was performed as described previously^30^.

### SDS polyacrylamide gel electrophoresis and Western blotting

Immunoprecipitated NPC1-Flag was denatured in Laemmli buffer containing 100 mM DTT for 5 min at 95 °C. Proteins were separated on 8% polyacrylamide gels and transferred to PVDF membranes. The membranes were blocked for 30 min in 5% milk in 50 mM Tris buffer pH 7.6 with 150 mM NaCl and 0.05% Tween 20 (TBST) at room temperature (RT). The membranes were incubated with mouse anti-Flag antibody 1:5000 for 1 h in 2% milk in TBST at RT followed by goat anti-mouse antibody conjugated to horseradish peroxidase 1:5000 for 1 h in 2% milk in TBST at RT. Protein bands were visualized as described before in^29^ with a ChemiDoc XRS System (Bio-Rad, California, USA).

### Confocal fluorescence microscopy

COS-1 cells were seeded on coverslips and transfected for 48 h using DEAE dextran. Transfected cells were fixed with 4% paraformaldehyde and quenched with 50 mM NH_4_Cl. Cells were then incubated for 2 min in cold methanol, washed with PBS at RT and permeabilized with PBS containing 0.5% saponin for 10 min at RT. Following permeabilization, cells were incubated with 1% BSA in PBST (PBS + 0.1% Tween 20) for 30 min. Immunostaining was performed using either mouse anti-Flag (1:200) or rabbit anti-Flag (1:200). To analyze co-localization, rabbit anti-calnexin (1:25), mouse anti-GM130 (1:25) and mouse anti-Lamp2 (1:25) antibodies were used to stain ER, Golgi and lysosomes respectively, followed by secondary antibody anti-rabbit-Alexa568 (1:500) and anti-mouse-Alexa488 (1:500). ProLong Gold Antifade with DAPI was used to visualize the cell nucleus and for mounting of the coverslips. The samples were examined by a Leica TCS SP5 confocal microscope with a HCPL APO 63 × 1.3 glycerol immersion objective. All immune fluorescence pictures have been sharpened using GIMP 2 program, using the same parameters.

### Isolation of detergent-resistant membranes

Transiently transfected COS-1 cells were solubilized at 4 °C for 2 h with Triton X-100 in 50 mM Tris buffer pH 7.6 with 150 mM NaCl. Discontinuous sucrose gradients were performed (1 ml 80% w/v sucrose, 1 ml lysate in 40% w/v sucrose, 7 ml 30% w/v sucrose and 1 ml 5% w/v sucrose). Gradients were centrifuged at 4 °C and 100,000 × g for 18 h using a Beckman-centrifuge equipped with a SW40 rotor. Ten fractions were collected from top to bottom at 4 °C. Fractions 1–3 are the floating fractions and considered as lipid rafts (LR) that contain low-buoyant density cholesterol and sphingolipids. Fractions 8–10 are the non-floating fractions of the gradient and called the non-lipid rafts (NLR). The association of NPC1-Flag with LR and the distribution of the LR marker protein flotillin-2 were determined by SDS-PAGE. 35 μl of each fraction was mixed with Laemmli buffer and DTT then separated on 12% gels followed by immunoblotting with mouse anti-flotillin-2 and mouse anti-Flag antibodies. The biosynthetic form of the wild type NPC1 protein and the representative mutations from each class was investigated by immunoprecipitation of Flag-tagged NPC1 from LR fractions (fractions 1 and 2) and the NLR fractions (fractions 8 and 9) using anti-Flag antibodies followed by treatment with endo H as described previously^30^.

## Results

### Expression of tagged forms of NPC1 in mammalian cells

To assess the structural and biosynthetic features as well as the intracellular localization of wild type (WT) and mutants of NPC1 in mammalian COS-1 cells as a model cellular system, Flag-tagged chimeras of NPC1 species were constructed. The Flag-tag was utilized for cellular localization (for example ER, Golgi, lysosomes) as well as the biochemical monitoring of the biosynthesis and processing of NPC1 and its mutants. It was necessary to generate the tagged due to the lack of a specific and reliable anti-NPC1 antibody.

### Identification and intracellular localization of biosynthetic forms of wild type NPC1

**Figure 1:**
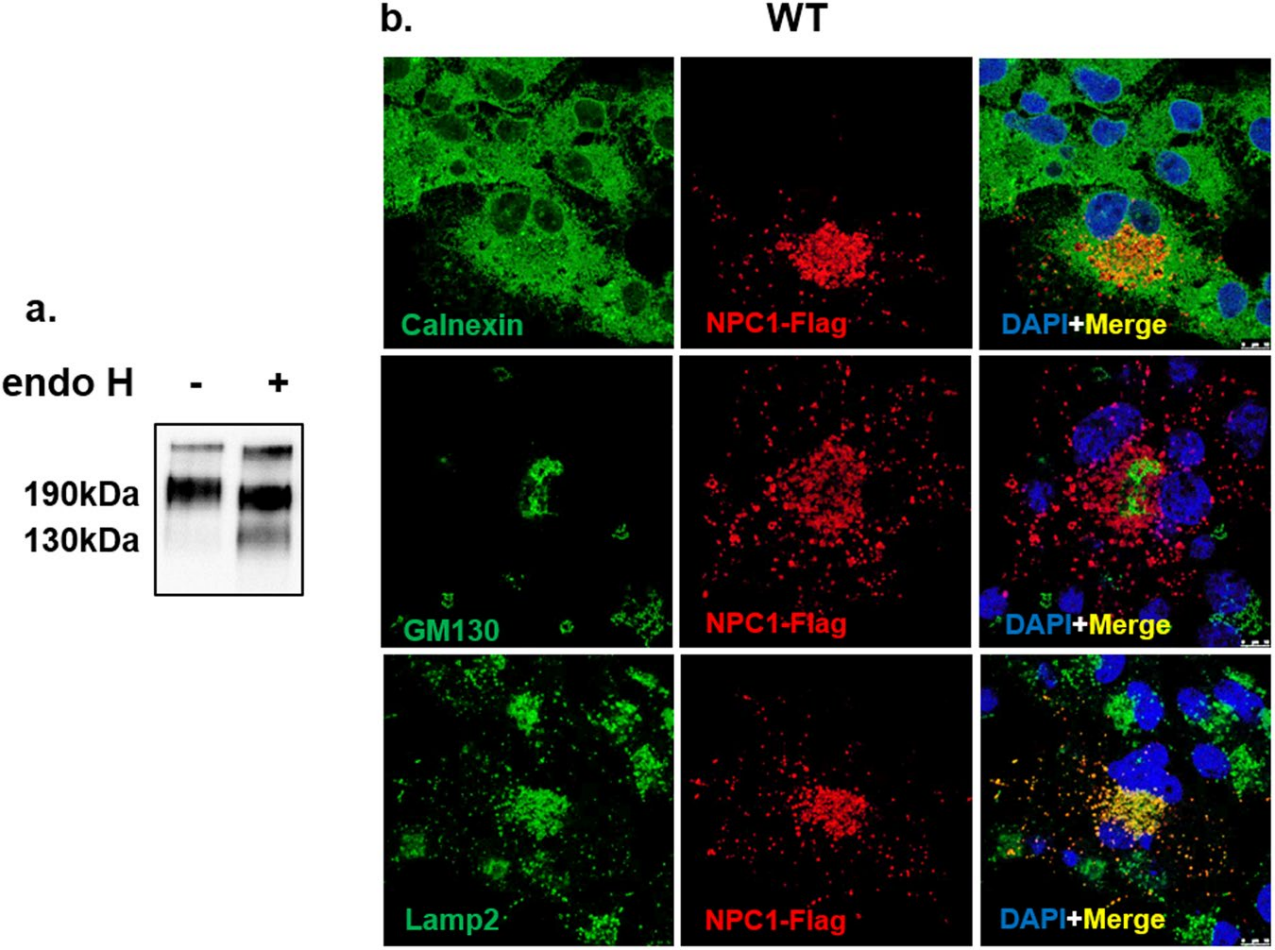
Biosynthetic forms of wild type NPC1. (**a**) COS-1 cells were transfected with cDNA encoding the wild type NPC1 tagged with Flag (WT). 48 hours after transfection the cells were lysed and treated with endo H to assess its biosynthetic forms. Wild type NPC1 appears as a single diffuse band comprising two biosynthetic forms that can be distinguished based on sensitivity or resistance towards endo H (referred to as NPC1c and NPC1h, i.e. the endo H-forms of complex and mannose-rich NPC1). (**b**) Immunostaining followed by confocal laser scanning microscopy of wild type NPC1 revealed the NPC1 predominantly in punctuate structures typical of the lysosomes as confirmed by its co-localization with Lamp2. Slight localization of NPC1 in the ER and the Golgi was also observed due its co-localization with calnexin and GM 130 (protein markers of the ER and Golgi respectively). Red: NPC1-Flag; blue: Dapi; green: ER/calnexin; Golgi/GM130; Lysosomes/Lamp2.

The biosynthetic forms of WT NPC1 were identified by immunoprecipitation of Flag-tagged NPC1 from cellular lysates using anti-Flag antibodies followed by endoglycosidase H treatment (endo H). Endo H cleaves mannose-rich carbohydrate chains that are covalently and cotranslationally added to the Asn-residues in the ER and the sensitivity of a glycoprotein isoform to this enzyme is compatible with its localization in the ER or at the early stages of the secretory pathway. In Fig. 1a WT NPC1 appears as a single diffuse band harboring two biosynthetic forms that can be discriminated by virtue of their sensitivity or resistance towards endo H. The mannose-rich form (NPC1_h_) that is normally located in the ER is cleaved to a smaller polypeptide of about 130-kDa upon endo H-treatment. Another form is the endo H-resistant complex glycosylated form (NPC1_c_) of an apparent molecular weight of 190-kDa. This form is processed from the NPC1_h_ in the Golgi and is ultimately trafficked to the lysosomes. The slight sensitivity of this form to endo H is indicative of a partial processing of a mannose-rich chain or the presence of a hybrid glycan chain. Immunostaining followed by confocal laser microscopy of WT NPC1 revealed the NPC1 predominantly in punctuate structures typical of the lysosomes as confirmed by its co-localization with Lamp2 (Fig. 1b). Slight localization of NPC1 in the ER and the Golgi was also observed as assessed by its co-localization with calnexin and GM130 (protein markers of the ER and Golgi, respectively).

### Identification of different mutant protein phenotypes of NPC1

Having identified the molecular forms and cellular localization of WT NPC1, we followed similar procedures to assess the biosynthetic forms of a group of mutants localized to various domains of the NPC1 protein (Table 1).

#### ER-located NPC1 mutants

**Figure 2:**
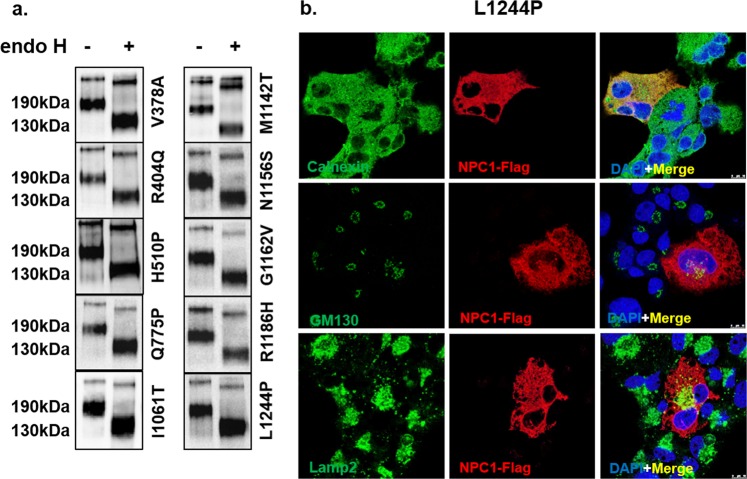
Biochemical characterization and cellular localization of the ER-located NPC1 mutants. (**a**) COS-1 cells were transfected with NPC1-Flag constructs harboring one of the following mutations: V378A, R404Q, H510P, Q775P, I1061T, M1142T, N1156S, G1162V, R1186H and L1244P. 48 hours after transfection the cells were lysed and treated with endo H and subjected to Western blotting using anti-Flag antibody. The mutants revealed complete endo H sensitivity and the mannose-rich form of the protein (NPC1h) was the only form detected in all mutations, no complex form of the protein (NPC1c) was detected. (**b**) The same mutants were analyzed by confocal microscopy. The NPC1 mutant (L1244P) was localized to the ER as assessed by the co-localization with the ER chaperone calnexin, while no co-localization was observed with the Golgi protein marker GM130 and the lysosomal marker Lamp2.

The first group of NPC1 mutants is represented by the mutations V378A, R404Q and H510P located in the second luminal loop, Q775P in transmembrane domain 7 (TMD 7), M1142T in TMD 10, G1162V and N1156S in TMD 11, L1244P in TMD 13, and finally R1186H and I1061T in the cytosolic domain. These mutations resulted in an NPC1 protein that was blocked intracellularly in the ER. As shown in Fig. 2a all of these NPC1 mutants without exception revealed a similar protein band pattern that was sensitive to endo H as assessed by the substantial shift in the size upon treatment with the endoglycosidase. There were no detectable endo H-resistant forms indicating that these NPC1 mutants are mannose-rich glycosylated. These results were corroborated by studying the intracellular localization by immunofluorescence. Figure 2b shows that all the NPC1 mutants were revealed in the ER as assessed by their co-localization with the ER chaperone calnexin, while no co-localization was observed with the Golgi protein marker GM130 and the lysosomal marker Lamp2 (Fig. 2b and electronic Supplementary Material 1).

#### Mutants revealing trafficking delay along the secretory pathway

**Figure 3:**
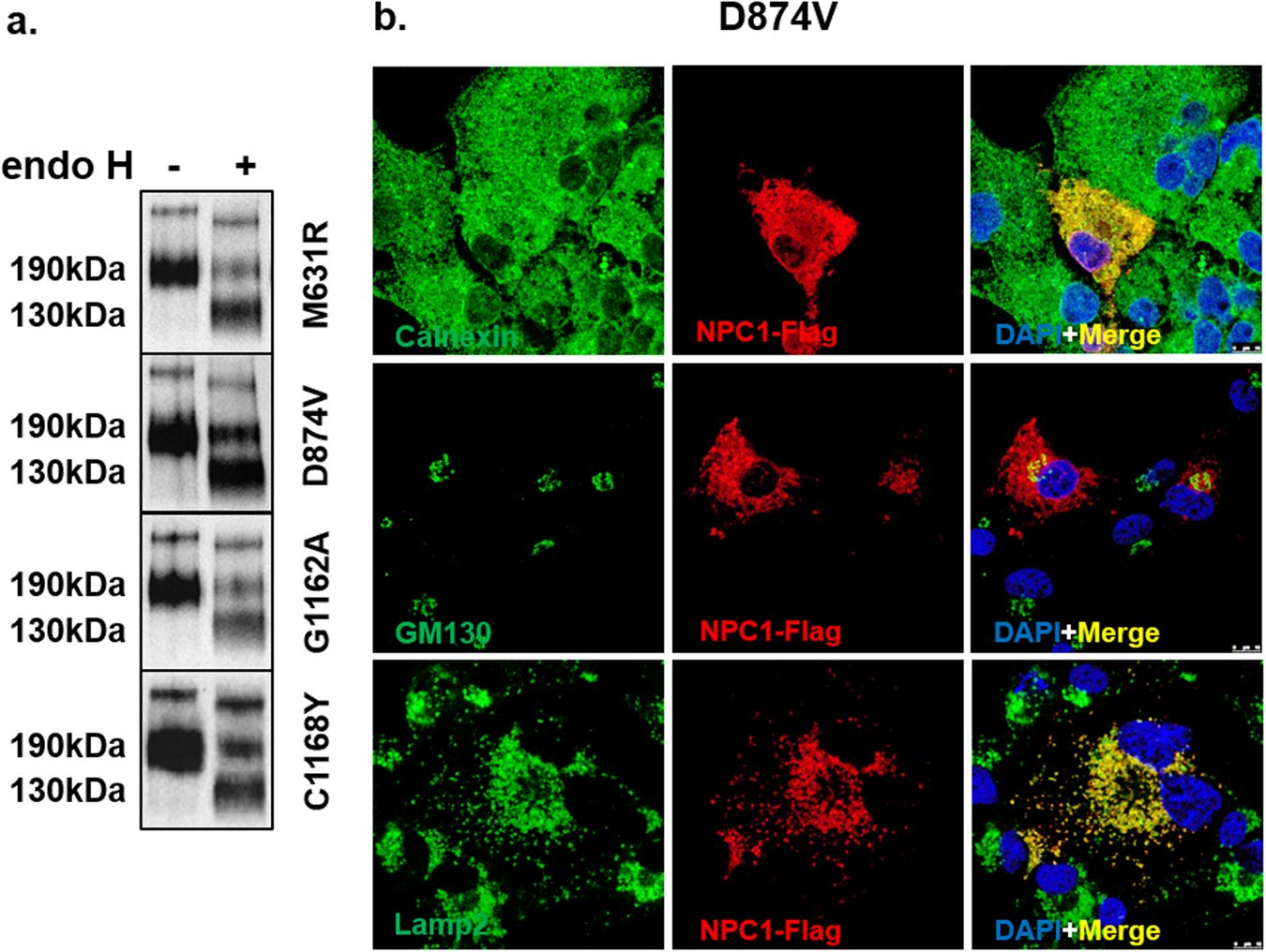
Biochemical characterization and cellular localization of NPC1 mutants exhibiting delayed trafficking. The same procedures were followed as in Fig. 2, except that the following mutations were analyzed: M631R, D874V, G1162A, and C1168Y. These mutant proteins revealed partial endo H sensitivity, whereby the mannose-rich (NPC1h) form and the complex glycosylated (NPC1c) form of the protein were both detected (**a**). Substantial co-localization of the NPC1 mutant (D874V) with calnexin and a partial co-localization with GM130 as well as with the lysosomal protein marker Lamp2 was clearly detectable (**b**).

The next group of mutants exhibited a trafficking delay of NPC1 mutants due to the mutations M631R in TMD3, G1162A and C1168Y in TMD11 as well as D874V in the luminal cysteine-rich loop. In these cases, NPC1 was capable of exiting the ER to the Golgi as demonstrated by the appearance of endo H-resistant protein forms of the NPC1 mutant proteins, which are compatible with the processing of NPC1 in the Golgi and the acquisition of complex type of N-linked glycans (Fig. 3a). The immunofluorescence images support the biochemical analyses. In fact, a partial co-localization of NPC1 mutants with GM130 as well as in the lysosomes with Lamp2 was clearly detectable in all the cases analyzed. The mutants co-localized also with the ER marker calnexin supporting the view that the trafficking out of the ER does not occur at a rate similar to the wild type NPC1 species (Fig. 3b and electronic Supplementary Material 2).

#### Mutants with wild type-like trafficking pattern

**Figure 4:**
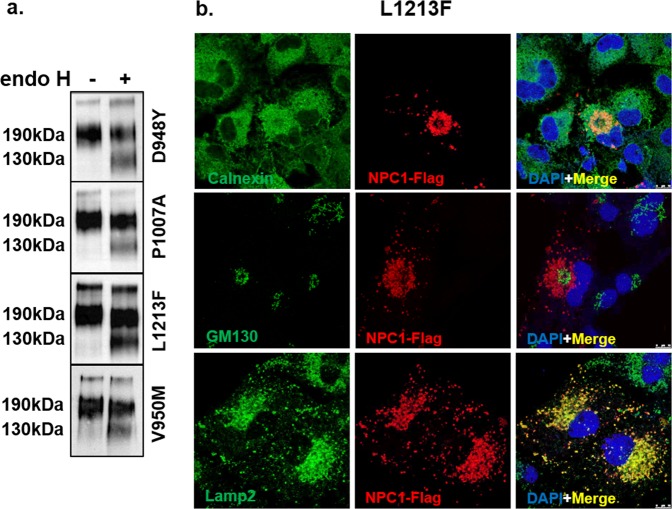
Biochemical characterization and cellular localization of NPC1 mutants trafficking similar to wild type NPC1. The same procedures were followed as in Fig. 2, except that the following mutations were analyzed: L1213F, D948Y, P1007A, and V950M. The biosynthetic forms of these mutants comprised mainly a complex glycosylated mature endo H-resistant form (NPC1c) (**a**) and the intracellular localization of the NPC1 mutant (L1213F) showed a partial co-localization with calnexin in the ER and a strong co-localization with the lysosomal marker Lamp2 (**b**).

The NPC1 mutants in this group, which resulted from the mutations L1213F in TMD12, P1007A and D948Y that located both in cysteine-rich loop, displayed protein biosynthetic forms similar to those of the wild type protein. As shown in Fig. 4a the biosynthetic forms of these mutants comprise mainly a complex glycosylated mature form NPC1_c_ and a low level of the mannose-rich form NPC1_h_. The intracellular localization of these mutants is similar to the wild type NPC1 and shows a strong co-localization with the lysosomal marker Lamp2. The co-localization with ER and Golgi protein markers that was detected in the partially trafficked mutants was not observed in this group of mutants (Fig. 4b and electronic Supplementary Material 3).

Altogether, our results provide a novel perspective of NP-C pathogenesis relevant to various phenotypes elicited by the different mutations in the gene encoding NPC1.

### Biosynthesis and turnover of wild type NPC1 and NPC1-mutants

**Figure 5:**
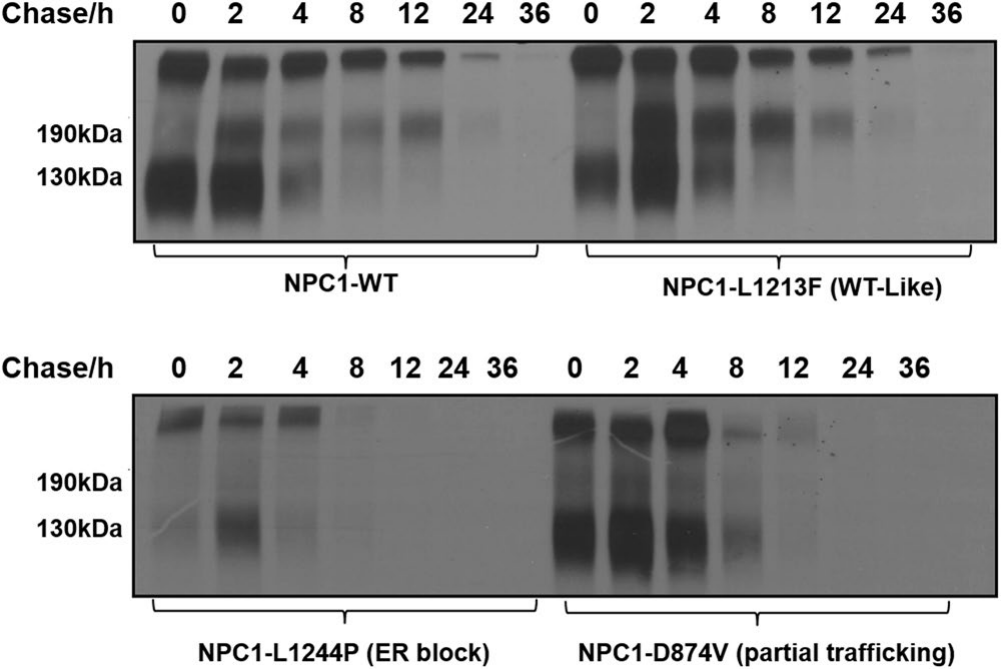
Biosynthesis and turnover of wild type NPC1 and NPC1 mutants representative of the three trafficking classes. COS-1 cells were transiently transfected with the cDNA encoding wild type NPC1-Flag or NPC1-Flag harboring the mutations L1213F, L1244P or D874V. 48 hours post-transfection the cells were biosynthetically labelled with ^35^S-methionine for 1 hour and chased for different hours (0, 2, 4, 8, 12, 24, 36). Immunoprecipitation of Flag tagged NPC1 was performed using anti-Flag antibody treated with endo H and subjected to SDS-PAGE on 8% slab gels.

The categorization of the NPC1 mutants into three classes that vary in their trafficking behavior and cellular localization raises questions relevant to the life cycle and turnover of these mutants and shedding thus light on another essential mechanistic element in the pathogenesis of NPC1-relevant NP-C. For this, the turnover rates of the mutants were compared with those of the wild type protein in pulse-chase experiments. The results of these studies revealed substantial variations, not only between the mutants and the wild type, but also between different mutants. These variations are directly associated with the localization of the NPC1 protein in the ER, Golgi or lysosomes. Thus, the NPC1 mutant due to the mutation L1244P disappears steadily throughout the chase time points and was no more detectable after 8 h of chase. By contrast, wild type NPC1 was still persistent, predominantly in its complex glycosylated form at the same time point. The wild type-like trafficked mutants revealed similar turnover rates. In fact, the mutant NPC1-L1213F was still present at 12 h as the wild type. Finally, the partially trafficked NPC1-D874V mutant was degraded at a lower rate than the ER-blocked NPC1-L1244P, but at a higher rate than the wild type (Fig. 5).

It is likely therefore that the variations in the turnover rates of the NPC1 mutants, their cellular localization and trafficking behavior can be associated with the level of impaired lipid trafficking itself with subsequent implications on the occurrence of clinical symptoms and their severity.

### Phenotypic variations are associated with alterations in the mode of interaction with the membrane

**Figure 6:**
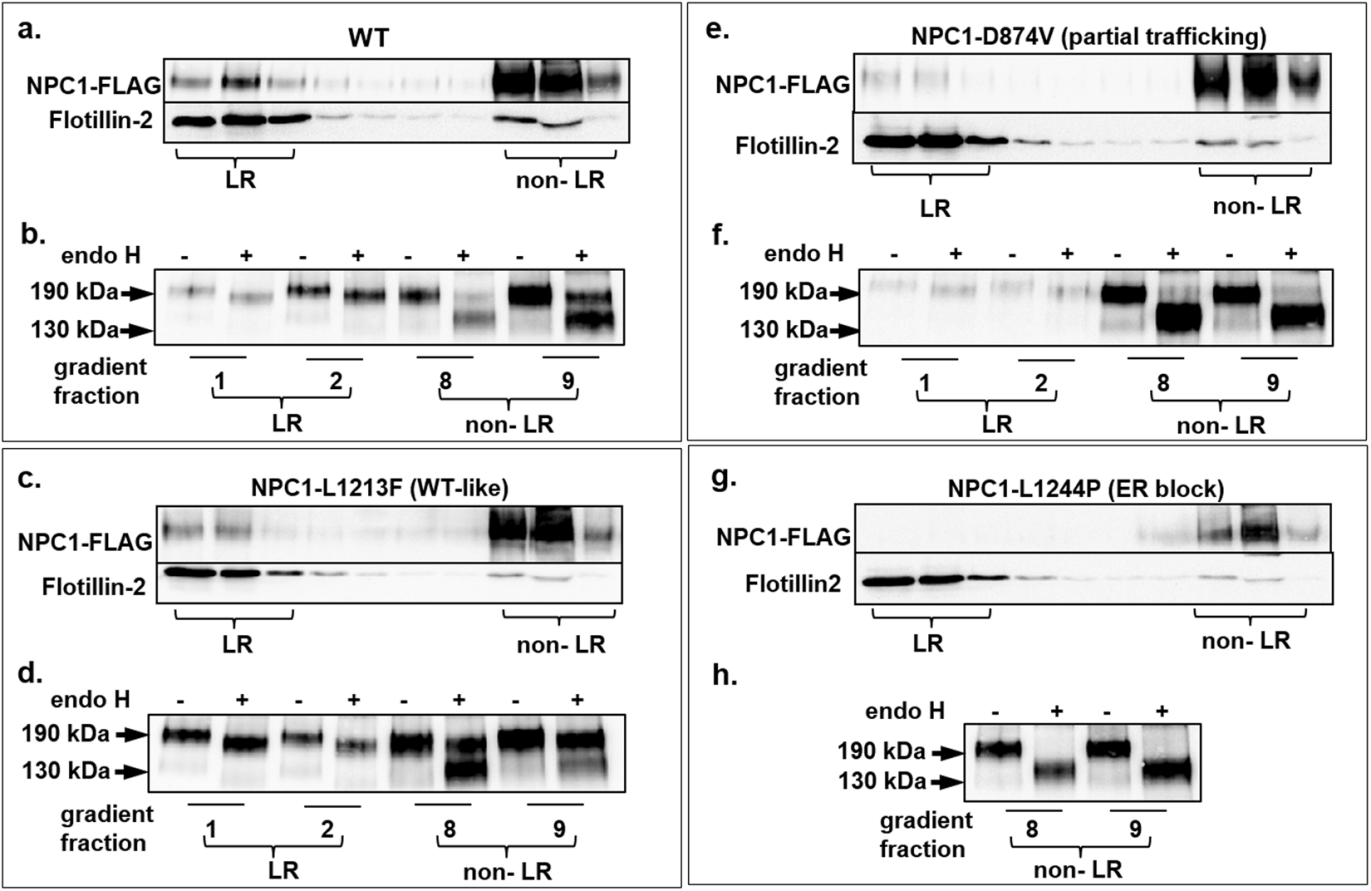
Mode of association of wild type NPC1 and mutants with membrane microdomains. Wild type NPC1-Flag **(a**,**b)** and NPC1-Flag mutants **(c**–**h)** representative of the three classes (L1213F, D874V and L1244P) were expressed in COS-1 cells. Triton X-100-cellular extracts were loaded onto a sucrose gradient and centrifuged at 4 °C and 100,000 g for 18 hours. Ten fractions were collected and analyzed by Western blotting. **(a)** Wild type NPC1 was found in the top three floating fractions co-localizing with flotillin-2, a typical lipid rafts marker. (**b**) The upper two floating fraction (lipid rafts-containing) and fraction 8 and 9 (soluble non-lipid rafts) were immunoprecipitated with anti-Flag antibody, followed by endo H treatment and Western blotting. The blot shows that the complex glycosylated endo H-resistant form of NPC1 (NPC1c) is the exclusive form of NPC1 that is associated with lipid rafts, while the soluble fractions contained predominantly the mannose-rich endo H-sensitive form (NPC1h) and to a lesser extent the mature NPC1 species. (**c**) and (**d**) represent similar experiments to A and B respectively but with L1213F. Note that more complex glycosylated-endo H-resistant NPC1-L1213F was found in the soluble non-rafts fraction as compared to wild type NPC1; (**e**) and (**f**) are similar experiments to A and B respectively but with D874V and finally, (**g**) and (**h**) are similar experiments to A and B respectively but with L1244P.

NPC1 is a multispan membrane glycoprotein that harbors 13 transmembrane domains and interacts with sphingolipids- and cholesterol-enriched lipid rafts. Several of the mutations analyzed in this study are located in these transmembrane domains raising the possibility that these mutations affected the mode of interaction of NPC1 with the membrane, particularly with lipid rafts. Other mutations that are located in the luminal domain including the cysteine-rich loop may affect the folding and quaternary structure of NPC1 and possibly also the interaction of the mutants with the membrane. We therefore addressed the association of wild type NPC1 and the mutants with lipid rafts by utilizing sucrose gradient centrifugation of cells that have been solubilized with 1% Triton X-100. Figure 6a shows that NPC1 was found in the floating fractions of the gradient co-localizing with flotillin-2, a typical lipid rafts marker^31^. NPC1 was also found in the soluble fractions of the gradient. This is not unusual since the distribution of NPC1 in the gradient corresponds to all NPC1 glycosylated forms at steady state along the secretory pathway, i.e. mannose-rich glycosylated, intermediate glycosylated and complex glycosylated forms. We determined which biosynthetic form of NPC1 was associated with lipid rafts by immunoprecipitation of NPC1 from the top 2 floating fractions (lipid rafts-containing) and fraction 8 and 9 (soluble non-lipid rafts) and performing endo H treatment. Figure 6b shows that the complex glycosylated endo H-resistant form of NPC1 is the exclusive form of NPC1 that is associated with lipid rafts. The soluble fractions contained predominantly the mannose-rich endo H-sensitive form and to a lesser extent mature NPC1.

We further examined the lipid rafts distribution of NPC1 mutants and analyzed one representative NPC1 mutant from each of the three characterized classes.

The wild type-like NPC1-L1213F was associated with lipid rafts as wild type NPC1 (Fig. 6c and d). Nevertheless, unlike wild type NPC1, more endo H-resistant complex glycosylated NPC1-L1213F was found in the soluble non-lipid rafts fractions as compared to wild type NPC1 (Fig. 6d, fraction 9). This result is compatible with a variable distribution of the mutant in the membrane, likely due to physiochemical characteristics elicited by the mutation L1213F that is located in the transmembrane domain 12.

The mutant NPC1-D874V, representing the partially trafficked NPC1 was associated with lipid rafts since it was recovered in the floating fractions of the sucrose gradient albeit to a substantially lower extent than the wild type protein and correlating well with the reduced levels of complex glycosylated mature NPC1 forms in these mutants. Obviously, mature NPC1 mutants follow a similar pathway as the wild type protein to the lysosomes via association with lipid rafts (Fig. 6e and f).

Finally, the transport-incompetent mannose-rich glycosylated class of mutants exemplified by the mutant NPC1-L1244P, showed no association with lipid rafts, compatible with the results above that this mutant is localized in the ER, which do not contain cholesterol- and glycosphingolipid enriched microdomains (Fig. 6g). Endo H treatment confirmed the exclusive mannose-rich type of glycosylation of this mutant being found exclusively in the soluble non-lipid rafts fractions (Fig. 6h).

## Discussion

Over 400 mutations have been identified in the gene encoding NPC1 which account for almost 95% of the NP-C disease. Up to now, a large body of information on the genetic patterns of NP-C patients relative to the clinical disease severity has accumulated. However, it is still unclear how a mutation does affect the biosynthetic presentation as well as intracellular localization of NPC1. We therefore addressed the question whether mutations in the *NPC1* gene generate different protein phenotypes or classes that vary in their trafficking, intracellular localization and function, which may ultimately correlate with the pattern and severity of clinical symptoms in NP-C patients.

Altogether, the cell biology studies presented in this paper disclose a novel concept in the pathogenesis of NP-C that relies on the diversity of the trafficking phenotypes of the NPC1 mutants. Based on the transport behavior of NPC1 mutants along the secretory pathway, their association with membrane microdomains (lipid rafts) and cellular localization, three major classes of NPC1 mutants were characterized. It should be noted that overexpression of the mutants in COS-1 cells, which may exacerbate ER folding of the mutants, is not crucial in this classification, since normal expression levels of the mutants in NPC1-knockout CHO cells (CT43)^32^ resulted in essentially similar protein phenotypes (data are not shown). Many of the mutants analyzed were blocked in the ER as immature mannose-rich proteins, while others were either partially trafficked through the Golgi to the lysosomes or trafficked in a comparable fashion to wild type NPC1. Interestingly, a particular trafficking phenotype is not elicited by mutations confined to a particular domain in NPC1, since these protein trafficking phenotypes arise from mutations that are distributed over different regions of NPC1. Furthermore, the interaction of the NPC1 mutants with lipid rafts varies among the different mutants analyzed in this study, indicating that their trafficking pattern could be associated with the reduced or abolished trafficking of cholesterol out of the lysosomes. Lipid rafts occur as microdomains within biological membranes that are resistant to solubilization with non-ionic detergents such as Triton X-100^33,34^ or Lubrol^35^ and are enriched in cholesterol and sphingolipids. As expected, the ER-located NPC1 mutants do not associate with lipid rafts or detergent-resistant membranes since cholesterol or sphingolipids are not abundant in this organelle. On the other hand, the level of association of the partially-trafficked mutants with lipid rafts is low and correlates well with the level of maturation of these mutants. Remarkably and in contrast to the previous two classes, the correctly trafficked wild-type-like mutant NPC1-L1213F as well as the wild type NPC1 varied substantially in their association levels with lipid rafts as compared to the other two classes. Nevertheless, the mature forms of the wild type-like mutants revealed a higher solubility than their wild type counterpart, compatible with reduced levels of lipid rafts components in the cells overexpressing the mutant. Undoubtedly, membrane lipid analysis of cells overexpressing these mutants is required to support the hierarchical concept of trafficking. This will also help in assessing alterations and subsequent effects of the NPC1-trafficking phenotypes on global membrane transport, including endocytic and endosomal trafficking^36,37^. Nevertheless, the current study suggests a possible link between the three classes of the NPC1 trafficking phenotype mutants, with the heterogeneous disease pattern including neurological, visceral, or psychiatric manifestations, hence the severity of NP-C disease. However, examination of a larger number of mutants is required to make definite conclusions.

Recently, two main clusters of symptoms were identified for NP-C cases relative to age: for the cases ≤ 4years of age, one cluster comprised exclusively visceral symptoms, compared to another cluster that combined all other signs and symptoms. For the cases > 4years of age, each cluster comprised a mixture of visceral, neurological and psychiatric symptoms. The study reports that visceral symptoms were most common in patients with an age ≤ 4years. On the other hand, neurological symptoms showed a higher prevalence in patients with an age > 4years, with the exception of hypotonia and delayed developmental milestones^38^. Therefore, comparison of the biochemical phenotype of the mutants with the clinical picture of NP-C in these subgroups along similar procedures as those described in our study could help understand the various developmental forms of NP-C as well as their severity.

The biochemical analysis in this study reveals a clear classification of NPC1 mutants based on intracellular trafficking of NPC1 along the secretory pathway that could form the basis for the variable impairment of cellular cholesterol trafficking and help establish a possible correlation between the genotype and the clinical phenotype.

In fact, the data presented in Table 2 correlate the trafficking pattern with the biochemical phenotype of NPC1 mutants and the clinical onset of the symptoms from previous studies. Mutations that elicit a wild-type like trafficking pattern of NPC1, such as P1007A and V950M, are associated with a mild biochemical phenotype as well as adult onset of neurological symptoms. By contrast, NPC1 mutants that are either partially trafficked or are blocked in the ER are associated with a severe biochemical phenotype and a clinical onset of the disease occurring in infantile, late infantile or juvenile age, hence a more severe clinical phenotype. However, the limited sample presented in Table 2 is not sufficient to demonstrate a statistically significant correlation of genotype with disease severity.

**Table 2:** Genotype-phenotype correlation of different NPC1 mutations. The data were compiled based on a literature search.

Number	Mutation	Processing	Biochemical Phenotype	Clinical Phenotype	Cell line	Reference
1	P1007A	**WT-like**	mild	Adult	Homozygous	^4, 41^
2	D948Y	**WT-like**	ND	ND	ND	^42^
3	L1213F	**WT-like**	ND	Juvenile	Heterozygous with Y1088C	^43^
4	V950M	**WT-like**	mild	Adult	Homozygous	^44, 45^
5	M631R	**Partial trafficking**	severe	ND	Homozygous	^44^
			severe	Late infantile	Heterozygous with I1061T	^4^
6	D874V	**Partial trafficking**	severe	No neurological symptoms	Homozygous	^46^
7	C1168Y	**Partial trafficking**	severe	Late infantile	Homozygous	^44^
8	G1162A	**Partial trafficking**	severe	ND	Homozygous	^41, 45, 47^
9	V378A	**ER block**	severe	Adult without neurological symptoms	Heterozygous with I1061T	^4, 44^
10	R404Q	**ER block**	severe	ND	Homozygous	^46, 48^
			severe		Heterozygous with D874V	
11	H510P	**ER block**	severe	Late infantile	Homozygous	^43^
12	Q775P	**ER block**	severe	Severe infantile	Homozygous	^41, 47^
			severe	Severe infantile	Heterozygous with N1156S	^45^
13	I1061T	**ER block**	severe	Juvenile	Homozygous	^44^
			mild	Juvenile	Heterozygous with P1007A	^4^
			mild	Adult	Heterozygous with V950M	
14	M1142T	**ER block**	severe	ND	Heterozygous with G248V	^48^
15	N1156S	**ER block**	mild	ND	Homozygous	^49^
			severe	Juvenile	Heterozygous with I1061T	^47^
16	G1162V	**ER block**	severe	ND	Heterozygous with I1061T	^50^
17	R1186H	**ER block**	severe	ND	Homozygous	^46^
			severe	ND	Heterozygous with N1156S	^48^
18	L1244P	**ER block**	ND	Late infantile	Heterozygous with P1007A	^51^

Moreover, it is important to allude to the interaction between the different classes of NPC1 mutants and the resulting hierarchy of the biochemical as well as the clinical phenotype in compound heterozygous patients. For instance, the homozygosity of the ER-located NPC1-I1061T mutant elicits a severe biochemical phenotype, which could be attributed to degradation of misfolded NPC1 mutant in the ER via the ERAD pathway^39,40^. On the other hand, the combination of the NPC1-I1061T with either NPC1-P1007A or NPC1-V950M, both of which are normally intracellularly transported, resulted in a mild biochemical phenotype. This proposes the existence of a hierarchy among NPC1 mutants in which a wild type-like mutant, such as NPC1-P1007A, determines the overall biochemical pattern in the disease. Therefore, a potential interaction between two mutants in a homozygote or compound heterozygote background has to be taken into consideration when the genotype is correlated with the clinical phenotype.

In view of the high allelic heterogeneity of the disease and the plethora of different disease-causing mutations, a correlation of the molecular data with the onset of the neurological symptoms is difficult to make. Our study does not explain the heterogeneity in symptoms in patients with the same genetic mutations suggesting that the precise mutation is only partly responsible for the phenotype of the disease.

The identification of various trafficking phenotypes of NPC1 mutants is compatible with a multi-facetted pattern of NP-C and may help to establish a genotype/phenotype correlation for the common NPC1 mutants. This, in turn, can be used to fill the gap between genetic testing and disease severity. The benefit of this type of analysis is to achieve a more detailed and comprehensive understanding of the biochemical alterations in NPC1, correlating it to the pathogenesis of NP-C. This may allow quantitative assessment of the efficacy of different (future) therapeutic options, and hence propose a phenotype-based therapeutic approach for NP-C patients based on the classification of NPC1 mutants. Based on the high number of mutations and frequency of compound heterozygosity in NPC1 further studies are required to evaluate this concept.

## Supplementary information

Supplementary Figures

Supplementary full length blots
