# Supplementary material for: Different Niemann-Pick C1 Genotypes Generate Protein Phenotypes that Vary in their Intracellular Processing, Trafficking and Localization
Source: Sci Rep. 2019 Mar 28;9:5292. doi: 10.1038/s41598-019-41707-y (PMC6438969; doi:10.1038/s41598-019-41707-y)

SUPPLEMENTARY INFORMATION

Different Niemann-Pick C1 Genotypes Generate Protein Phenotypes that Vary in their Intracellular Processing, Trafficking and Localization

Hadeel Shammas, Eva-Maria Kuech, Sandra Rizk, Anibh M Das, Hassan Y Naim

SUPPLEMENTARY FIGURES

Captions for supplementary figures:

ESM 1. Cellular localization of the ER-located NPC1 mutants
COS-1 cells were transfected with NPC1-Flag constructs harboring one of the following mutations: V378A, R404Q, H510P, Q775P, I1061T, M1142T, N1156S, G1162V and R1186H**.** The mutants were analyzed by confocal microscopy. All NPC1 mutants were localized to the ER as assessed by the co-localization with the ER chaperone calnexin, while no co-localization was observed with the Golgi protein marker GM130 and the lysosomal marker Lamp2.

ESM 2. Cellular localization of NPC1 mutants exhibiting delayed trafficking
COS-1 cells were transfected with NPC1-Flag constructs harboring one of the following mutations: M631R, G1162A, and C1168Y**.** The mutants were analyzed by confocal microscopy. Substantial co-localization of all NPC1 mutants with calnexin and a partial co-localization with GM130 as well as with the lysosomal protein marker Lamp2 was clearly detectable.

ESM 3. Cellular localization of NPC1 mutants trafficking similar to wild type NPC1
COS-1 cells were transfected with NPC1-Flag constructs harboring one of the following mutations: D948Y, P1007A, and V950M. The mutants were analyzed by confocal microscopy. The intracellular localization of all NPC1 mutants showed a partial co-localization with calnexin in the ER and a strong co-localization with the lysosomal marker Lamp2.

ESM_1

NPC1 mutants that are blocked in the endoplasmic reticulum

Mutation N1156S

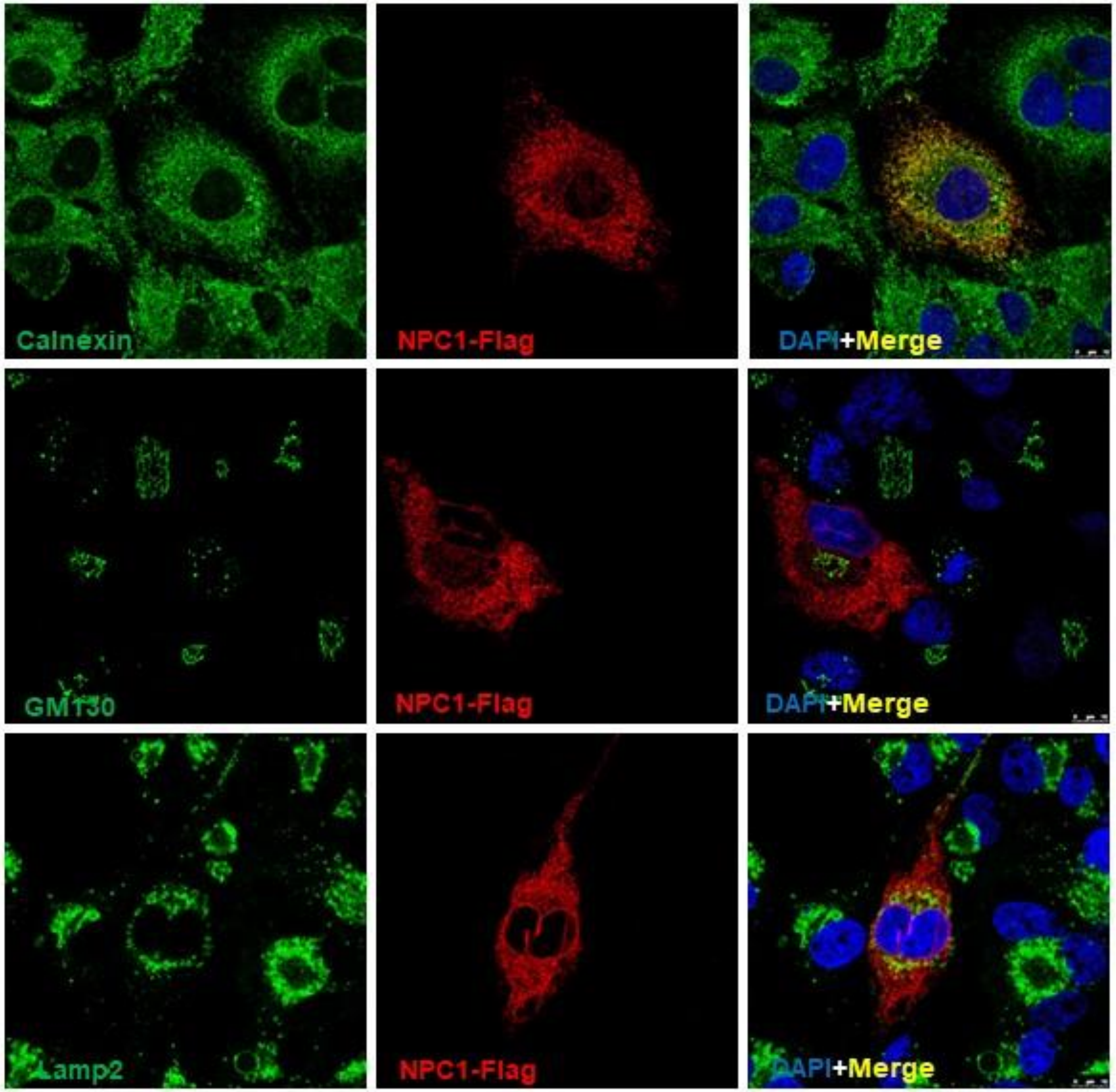

ESM_1

NPC1 mutants that are blocked in the endoplasmic reticulum

Mutation H510P

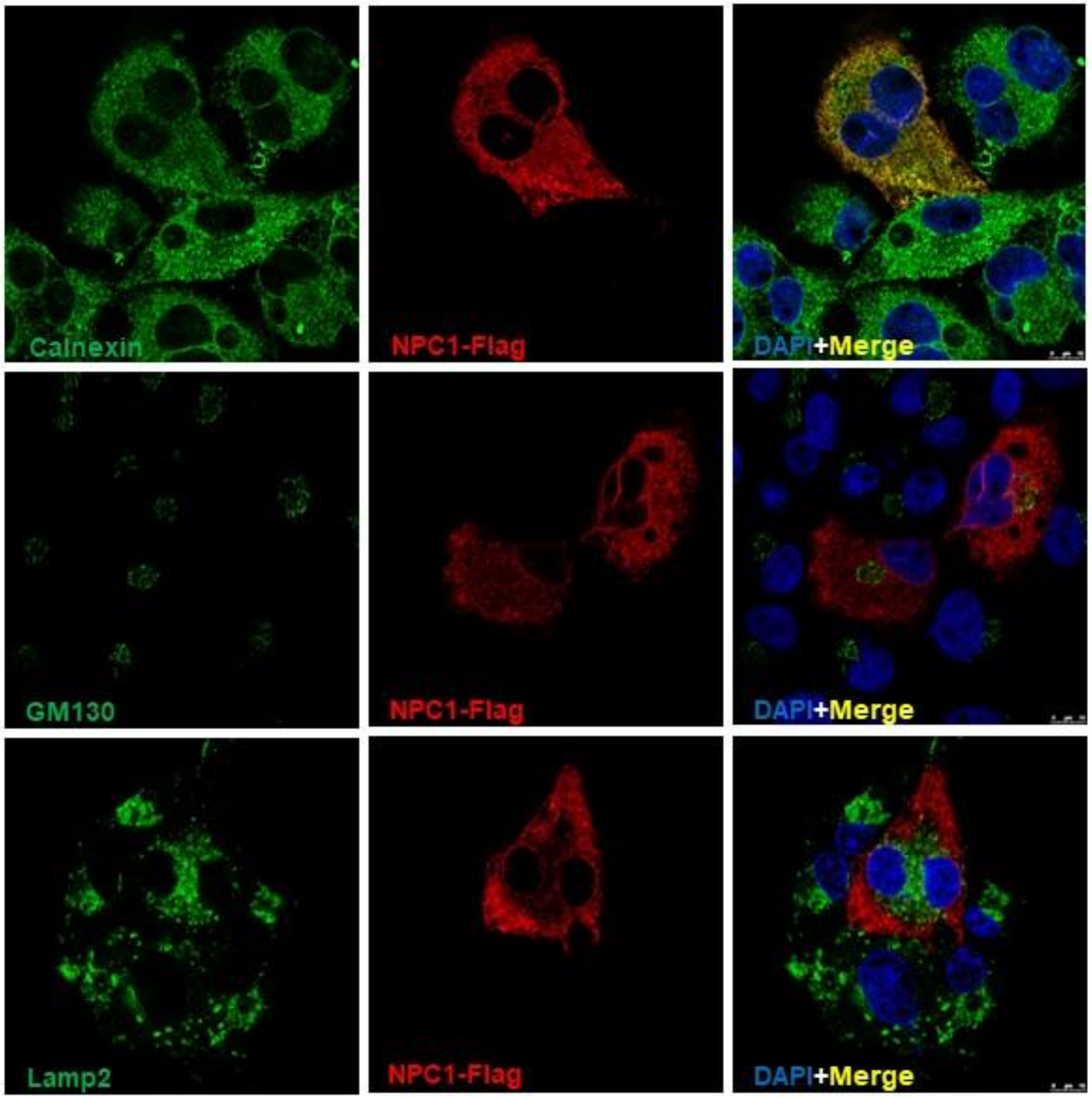

ESM_1

NPC1 mutants that are blocked in the endoplasmic reticulum

Mutation R404Q

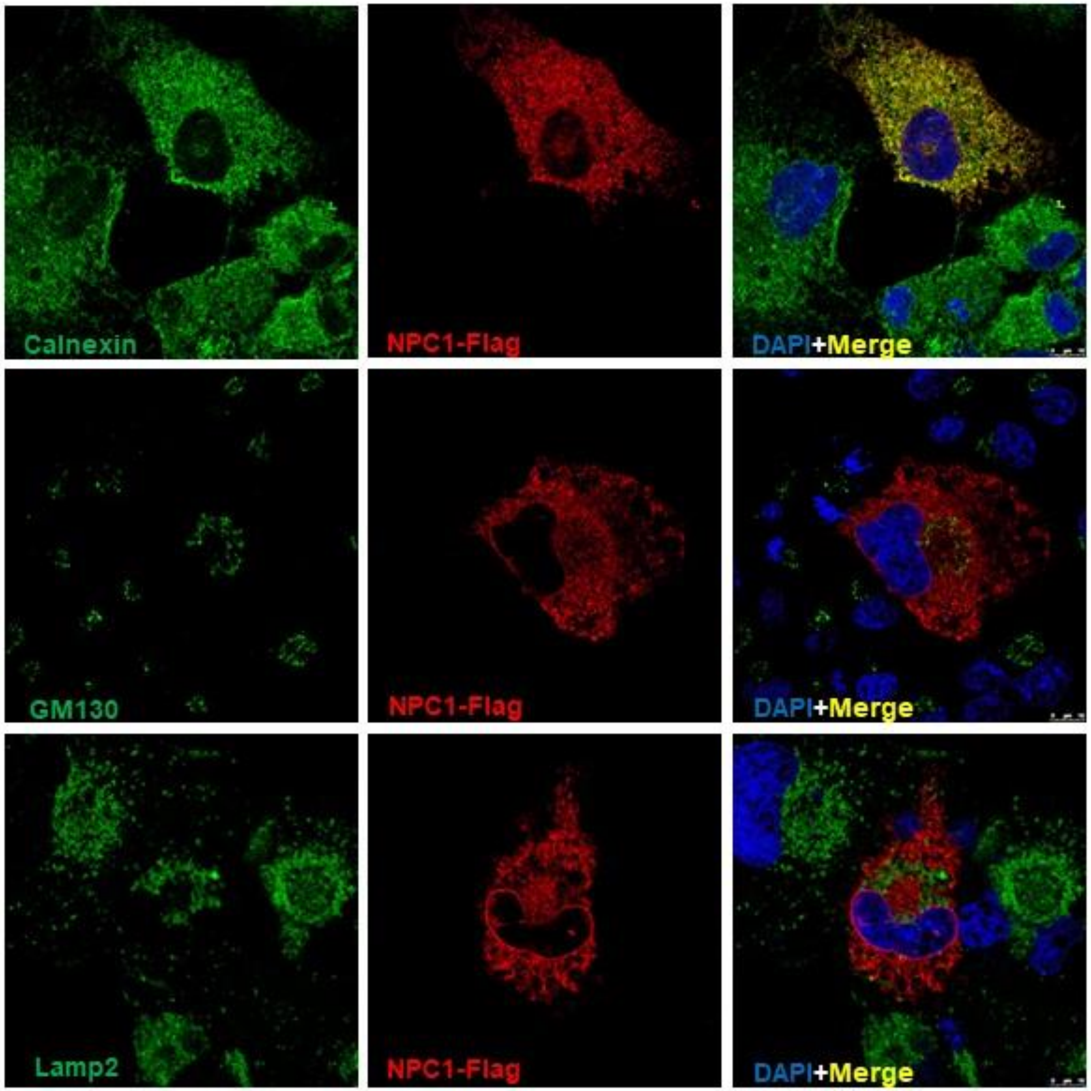

ESM_1

NPC1 mutants that are blocked in the endoplasmic reticulum

Mutation V378A

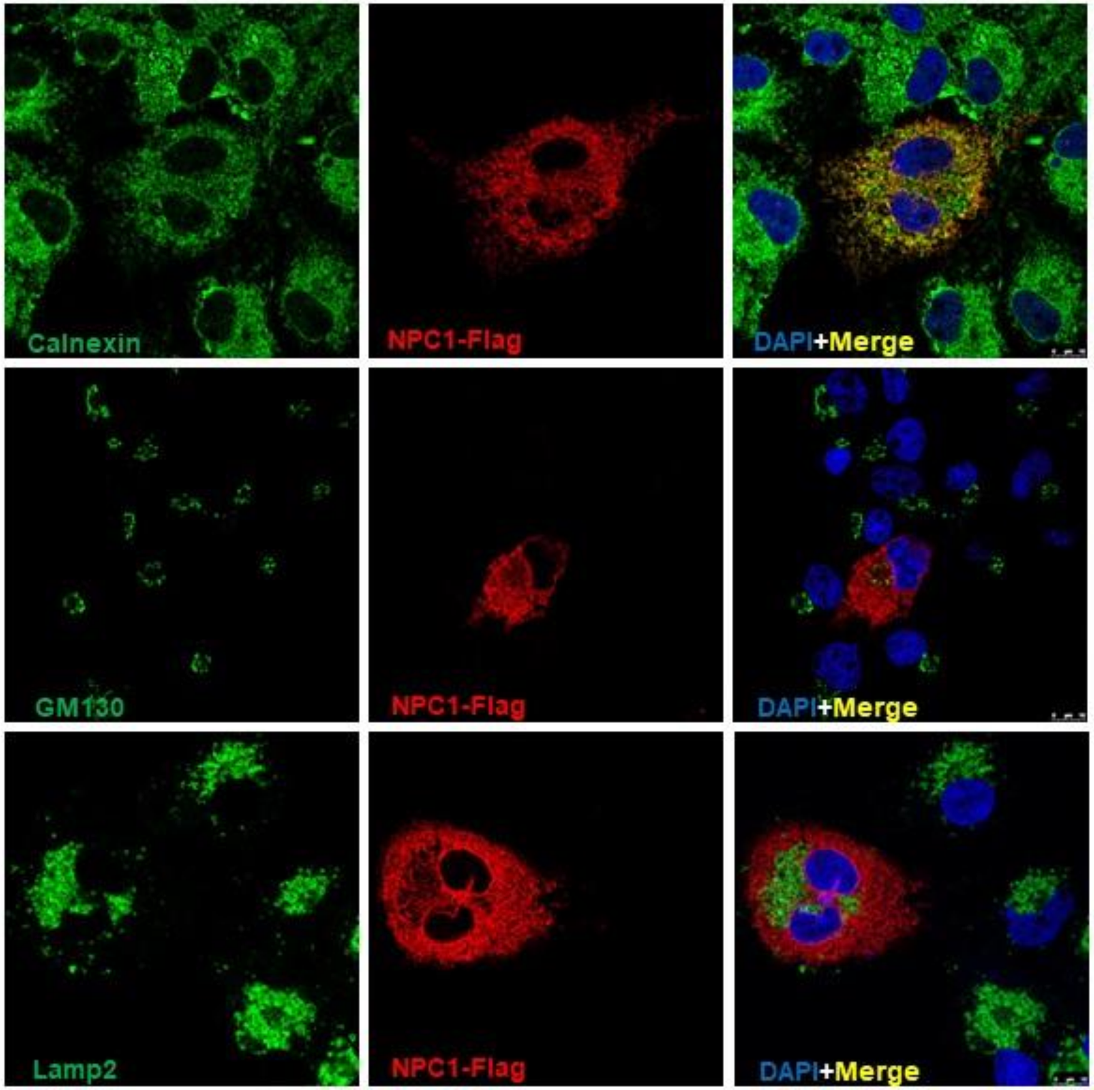

ESM_1

NPC1 mutants that are blocked in the endoplasmic reticulum

Mutation I1061T

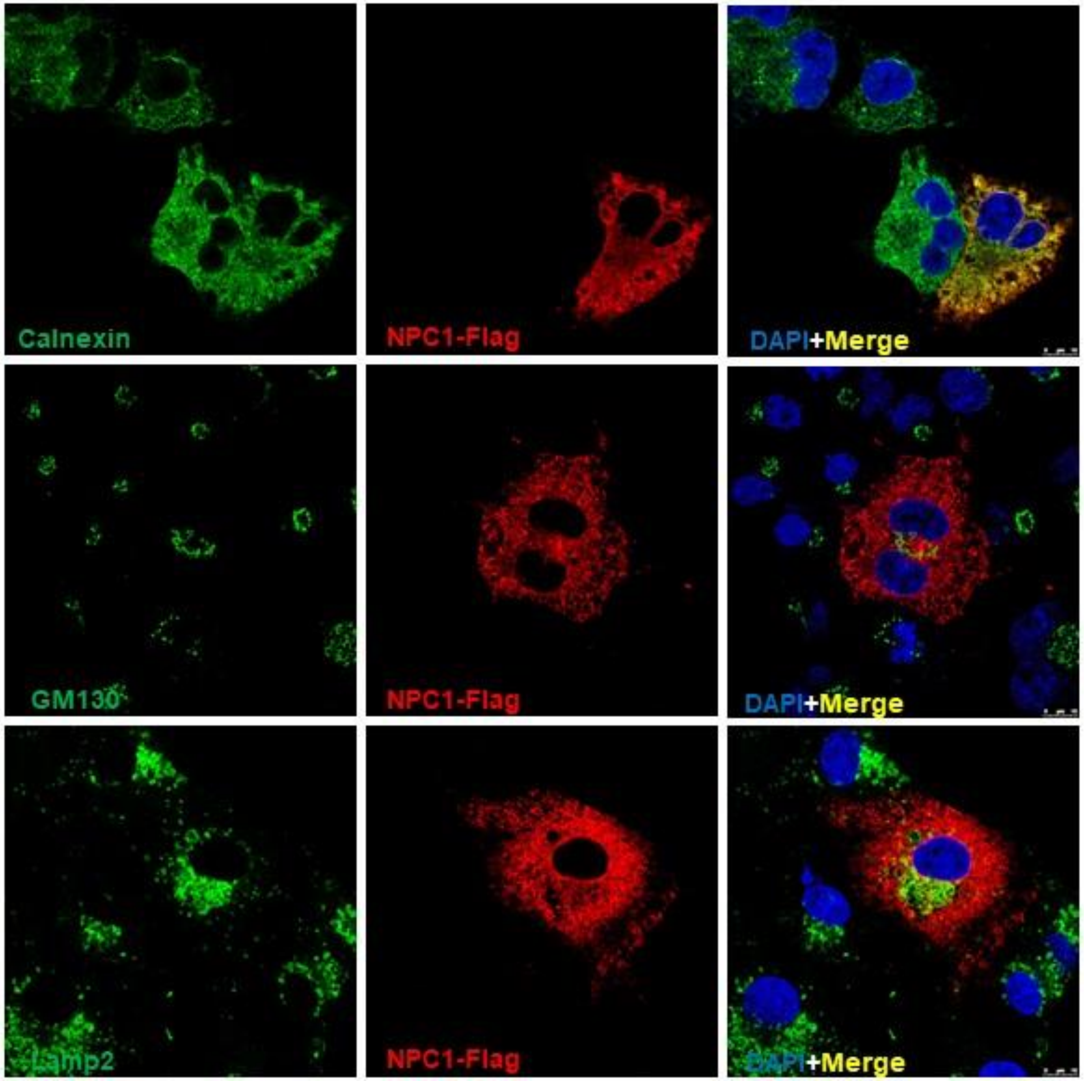

ESM_1

NPC1 mutants that are blocked in the endoplasmic reticulum

Mutation M1142T

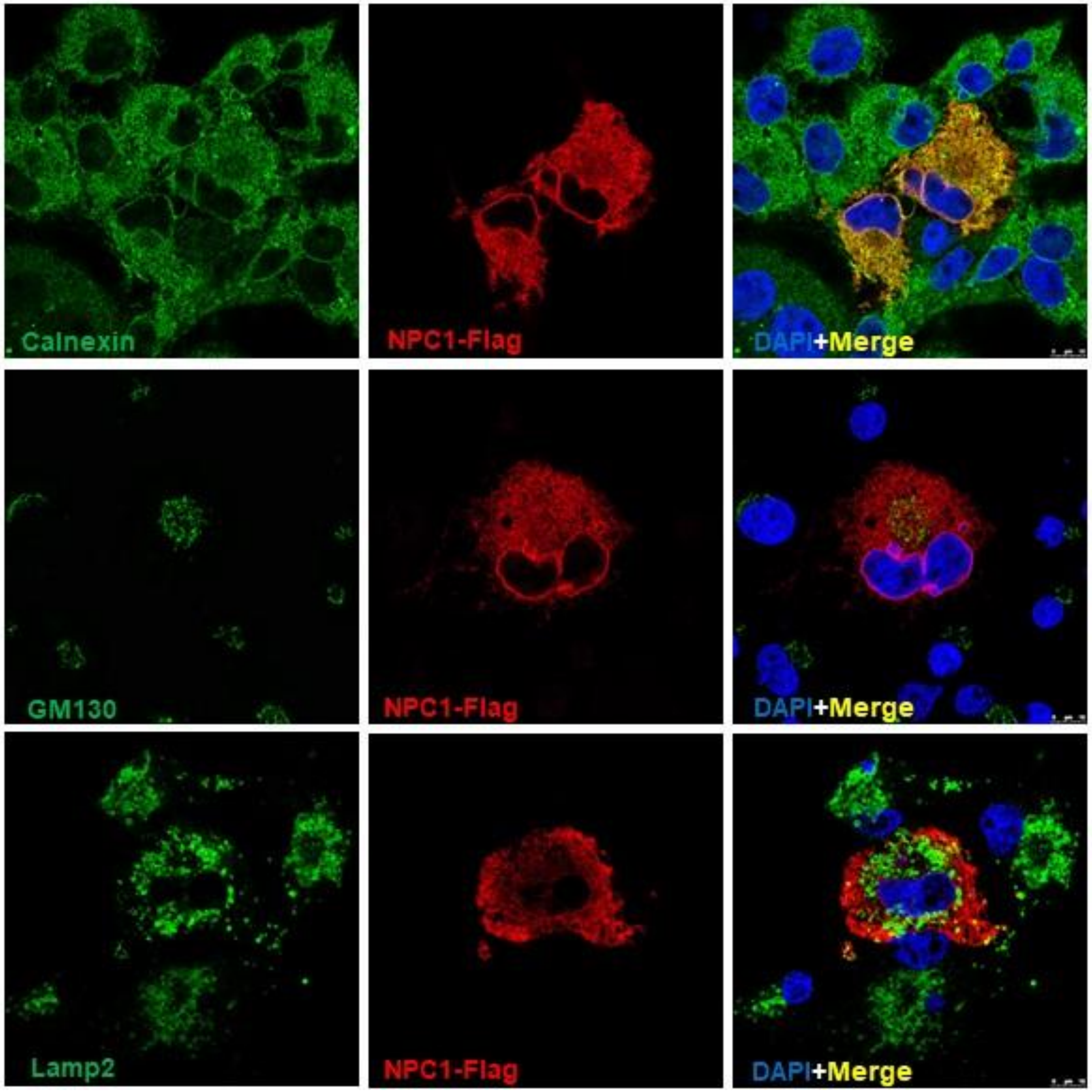

ESM_1

NPC1 mutants that are blocked in the endoplasmic reticulum

Mutation G1162V

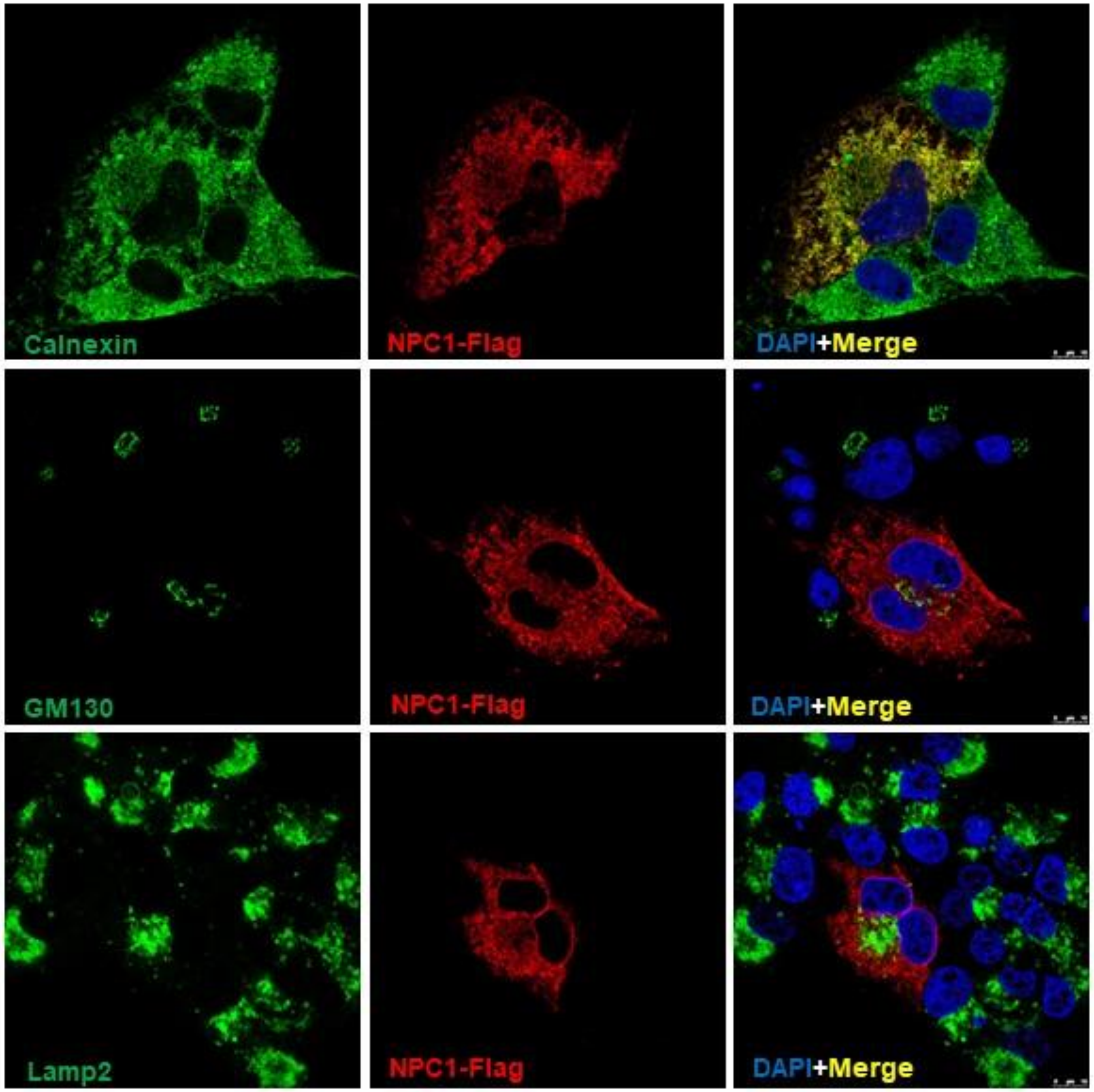

ESM_1

NPC1 mutants that are blocked in the endoplasmic reticulum

Mutation Q775P

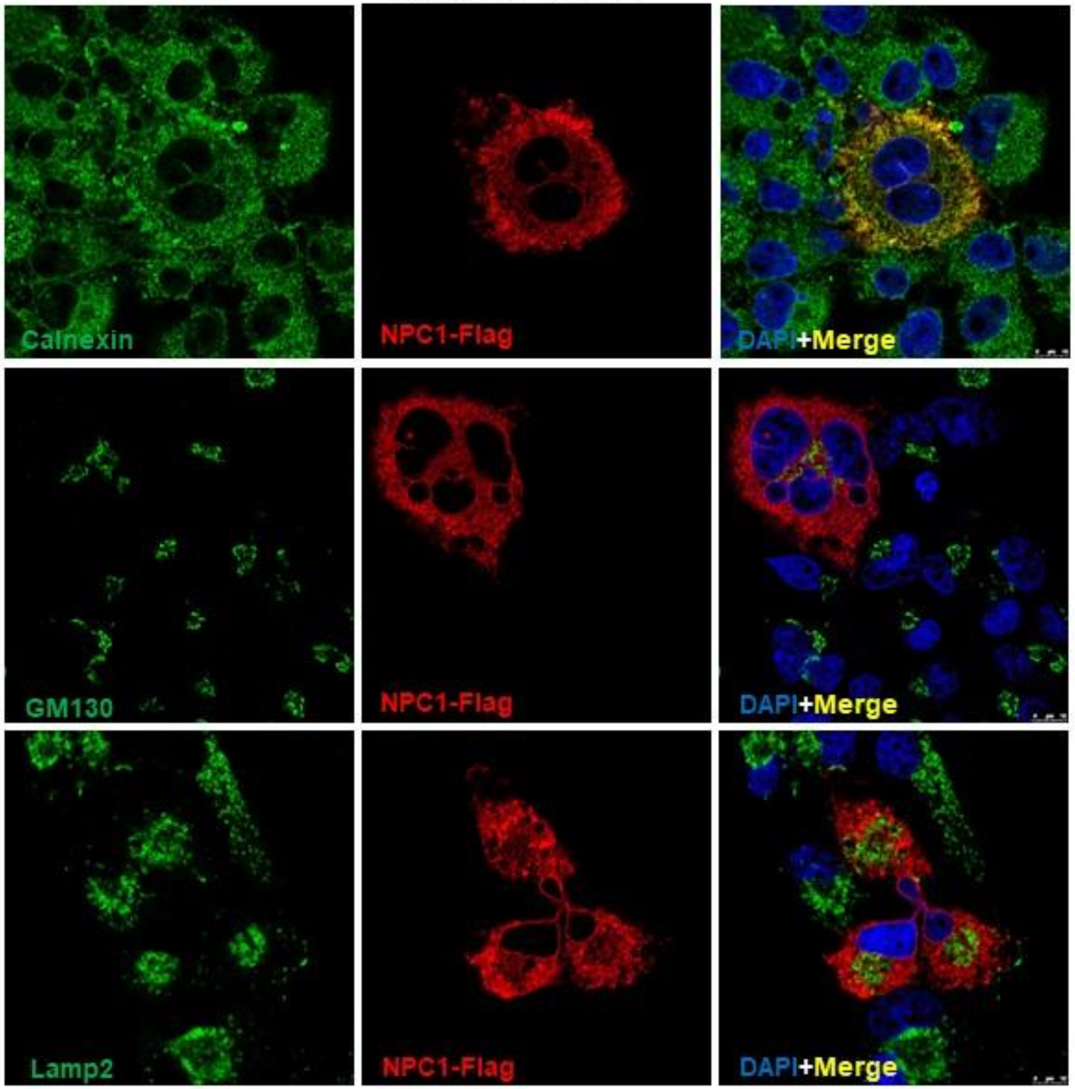

ESM_2

NPC1 mutants that are partially trafficked along the secretory pathway

Mutation C1168Y

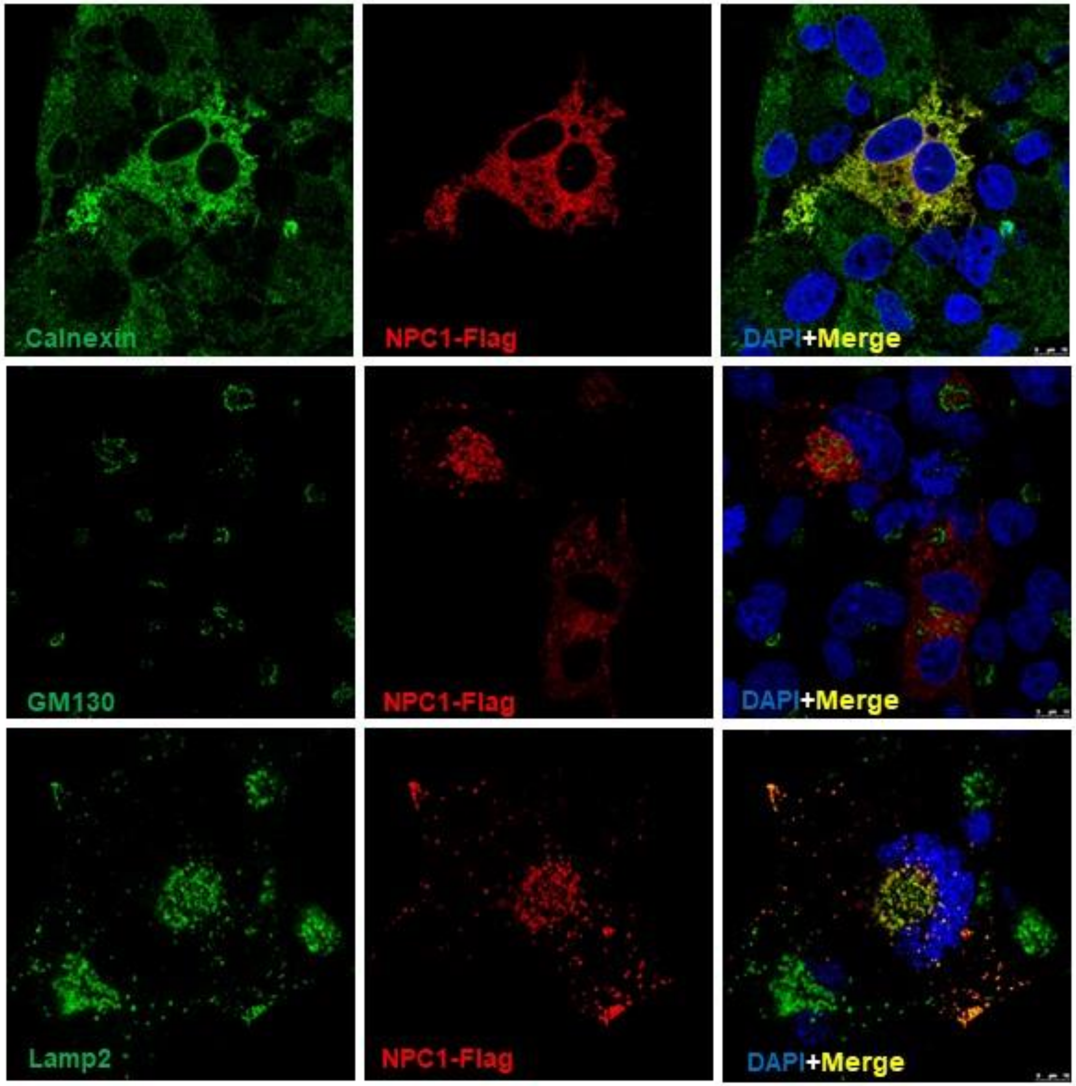

ESM_2

NPC1 mutants that are partially trafficked along the secretory pathway

Mutation M631R

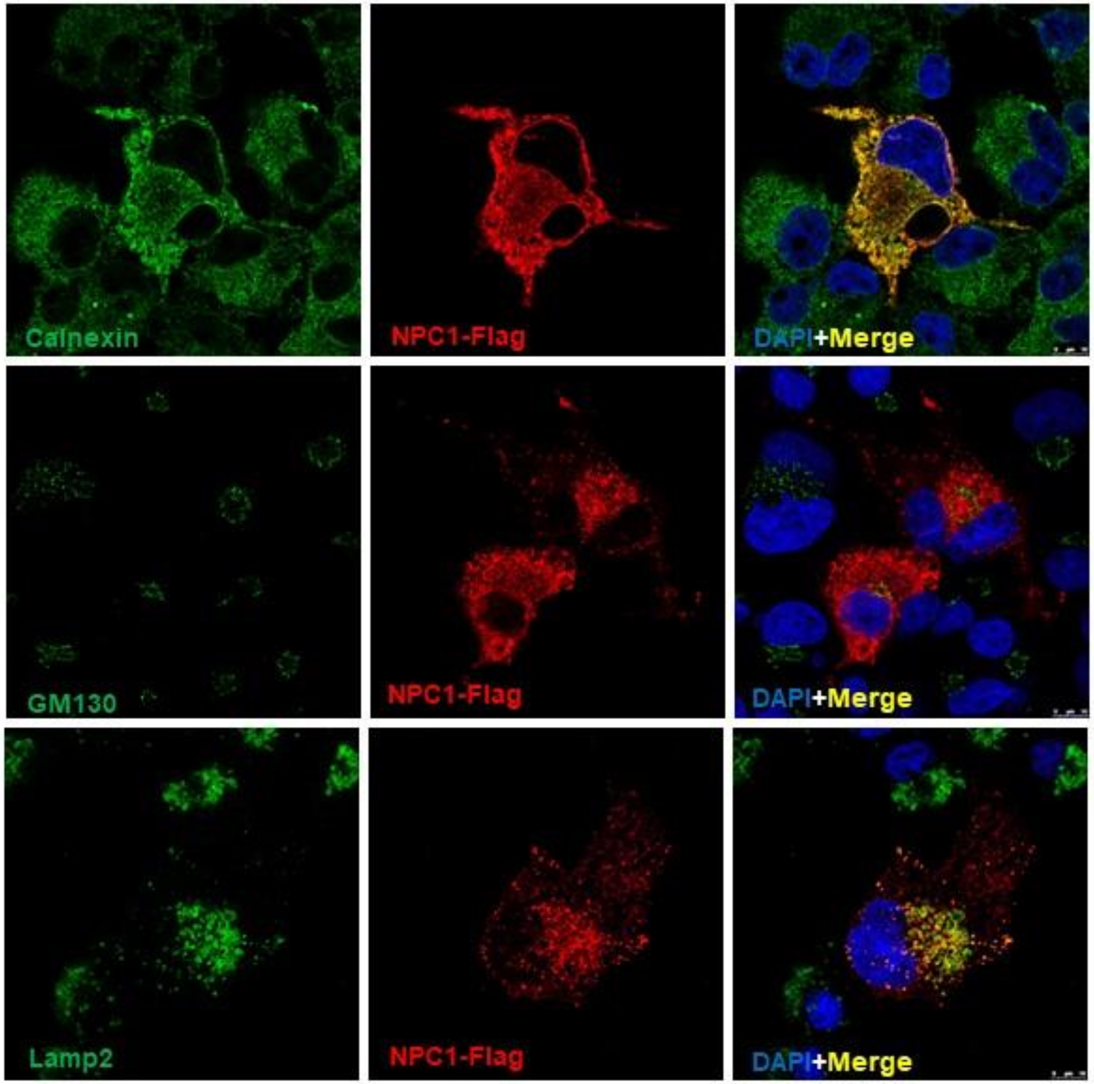

ESM_2

NPC1 mutants that are partially trafficked along the secretory pathway

Mutation G1162A

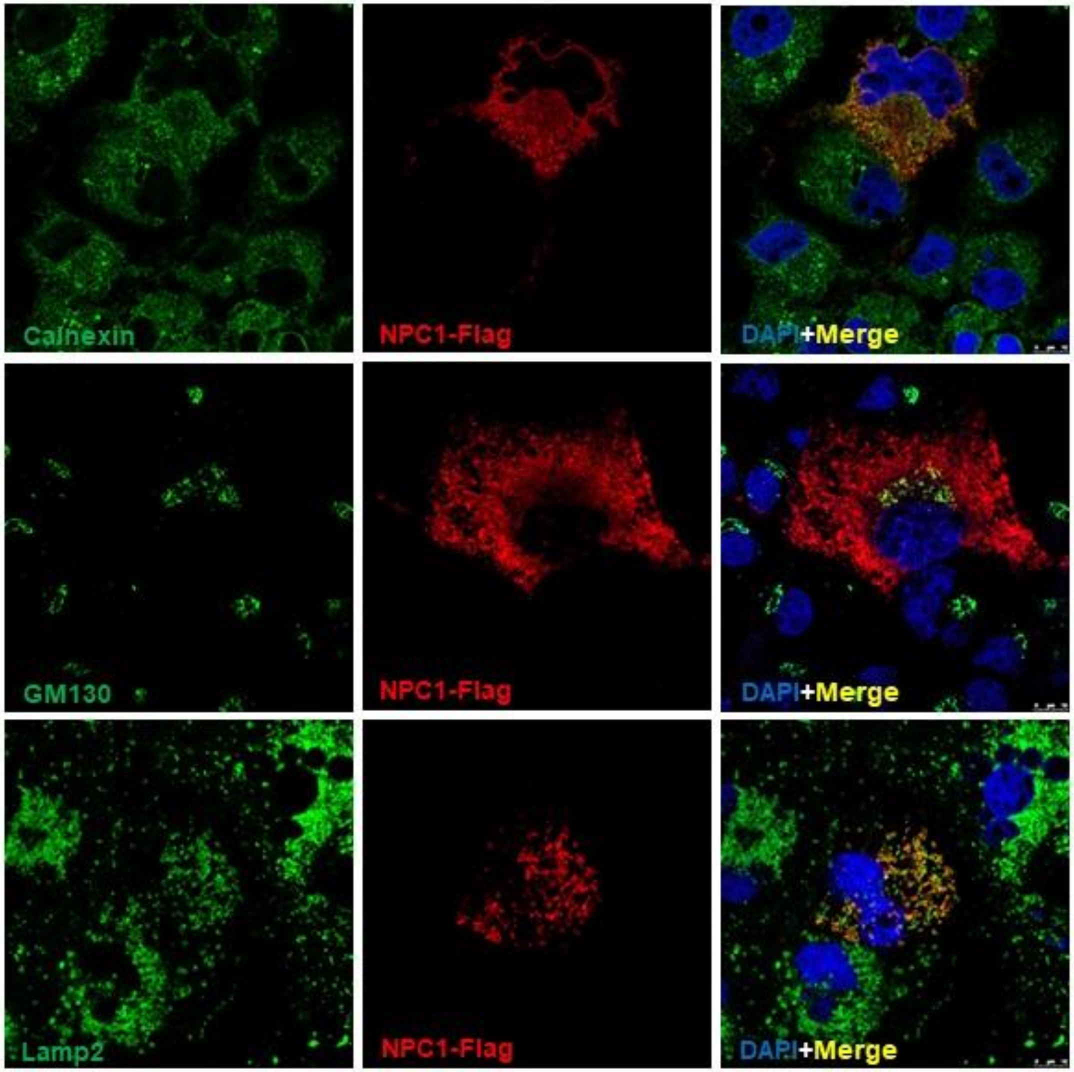

ESM_3

NPC1 mutants that are trafficked in a fashion similar to wild type NPC1

Mutation D948Y

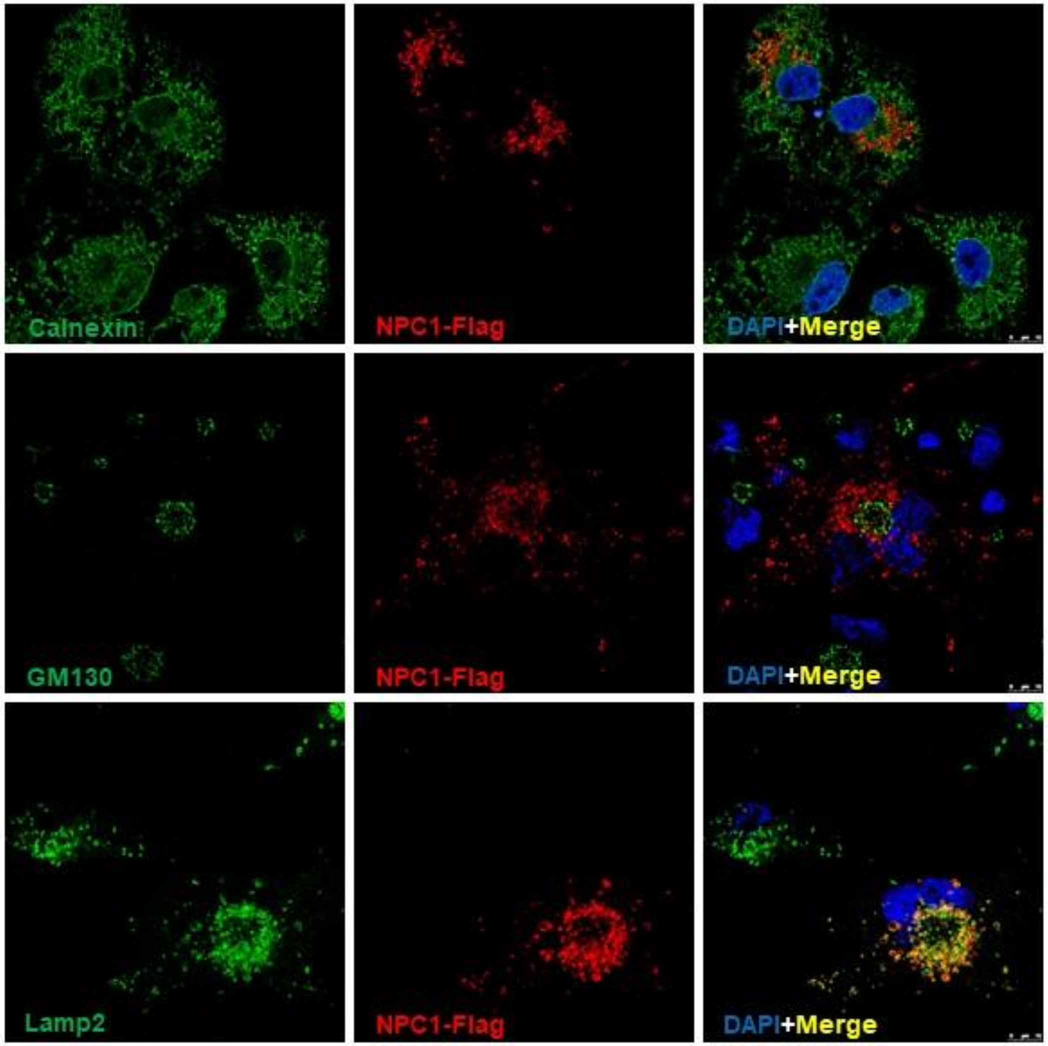

ESM_3

NPC1 mutants that are trafficked in a fashion similar to wild type NPC1

Mutation V950M

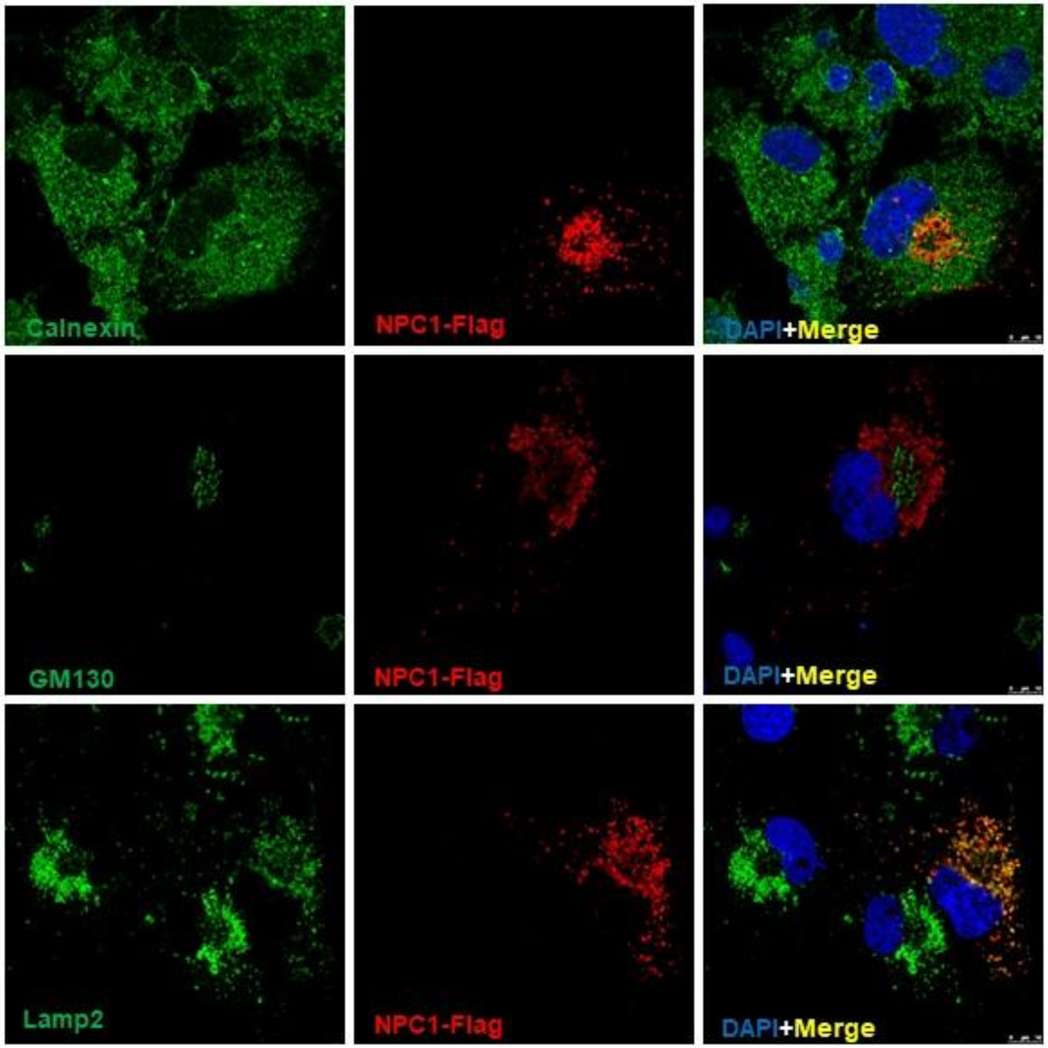

Supplement: Supplementary file 1 — Supplementary Figures [file 41598_2019_41707_MOESM1_ESM.pdf]
