# Supplementary material for: Different Niemann-Pick C1 Genotypes Generate Protein Phenotypes that Vary in their Intracellular Processing, Trafficking and Localization
Source: Sci Rep. 2019 Mar 28;9:5292. doi: 10.1038/s41598-019-41707-y (PMC6438969; doi:10.1038/s41598-019-41707-y)
Supplement: Supplementary file 2 — Supplementary full length blots [file 41598_2019_41707_MOESM2_ESM.pdf]

SUPPLEMENTARY INFORMATION

Different Niemann-Pick C1 Genotypes Generate Protein Phenotypes that Vary in their Intracellular Processing, Trafficking and Localization

Hadeel Shammas, Eva-Maria Kuech, Sandra Rizk, Anibh M Das, Hassan Y Naim

Full-length blots that are reported in the manuscript

Gel #2

WT #5G1162A #9 M631R #13 D874V

250kD
130kD

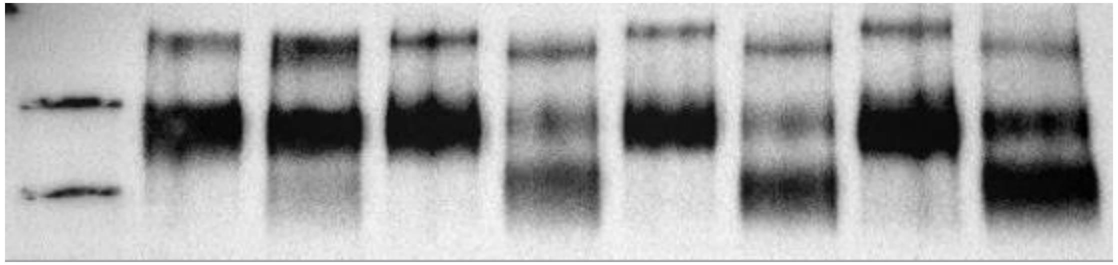

EndoH - + - + - + - +

Gel #3

WT #18C1168Y #17 R1186H #2 N1156S

250kD
130kD

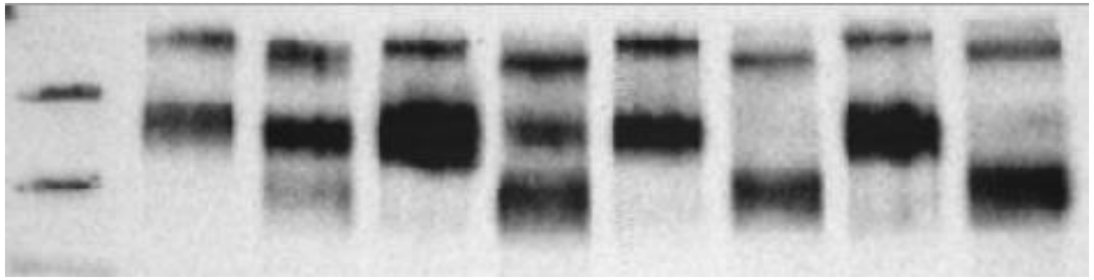

EndoH - + - + - + - +

Gel #4

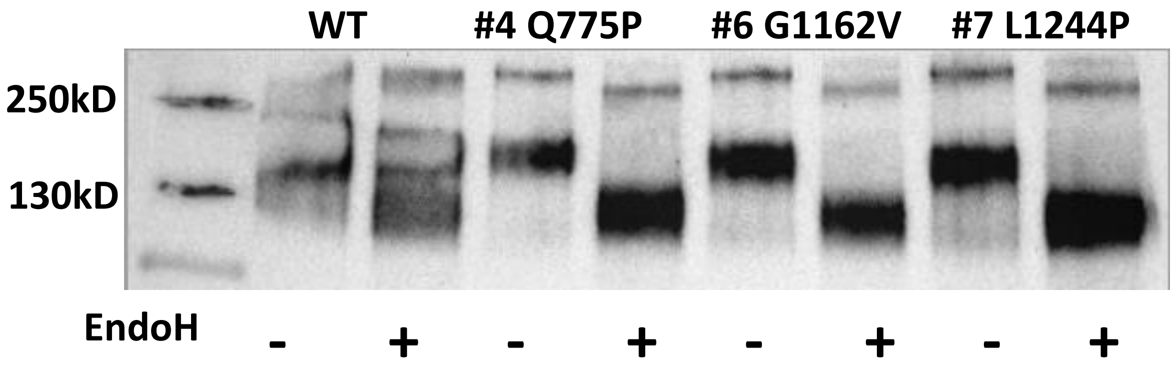
WT
#4 Q775P
#6 G1162V
#7 L1244P
250kD
130kD
EndoH
- + - + - + - +

Gel #5

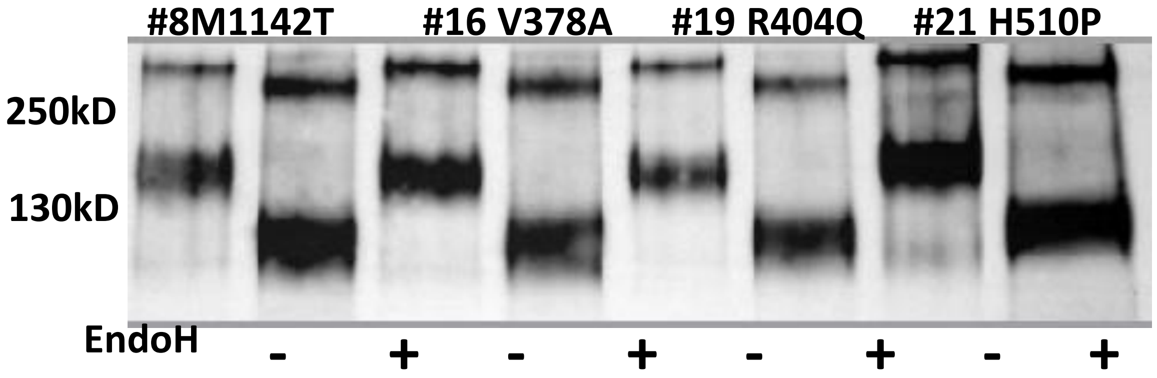
#8M1142T
#16 V378A
#19 R404Q
#21 H510P
250kD
130kD
EndoH
- + - + - + - +

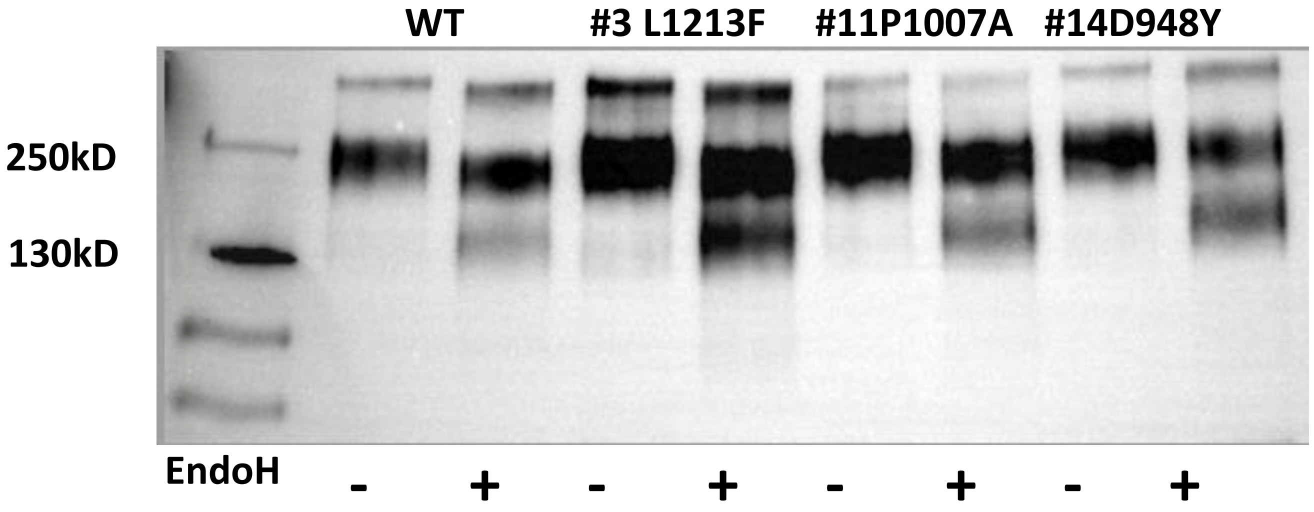
WT
#3 L1213F
#11P1007A
#14D948Y
250kD
130kD
EndoH
-
+
-
+
-
+
-
+

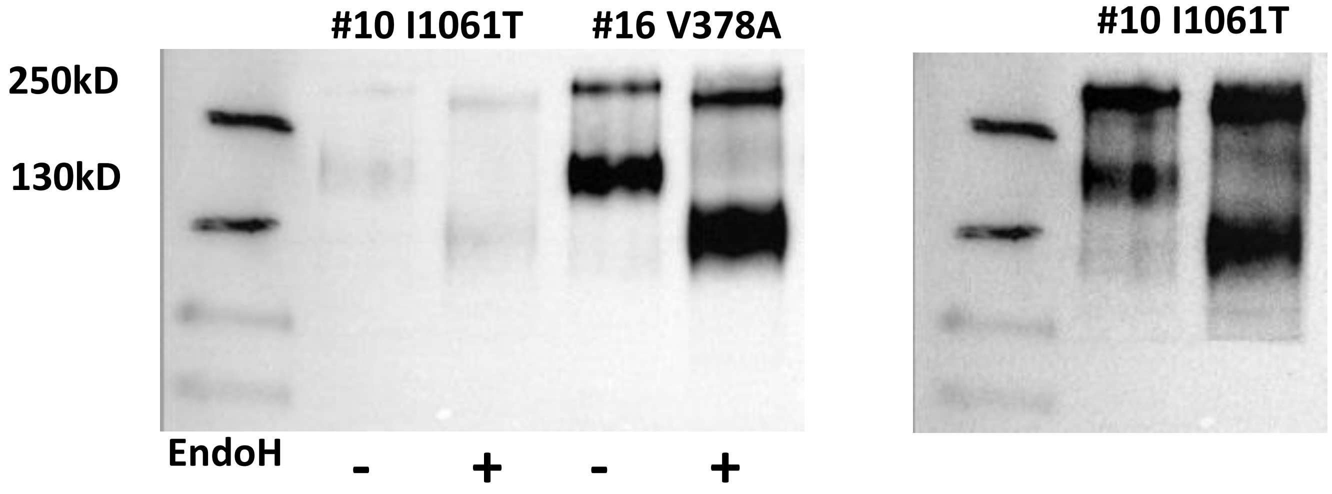
#10 I1061T
#16 V378A
#10 I1061T
250kD
130kD
EndoH
-
+
-
+

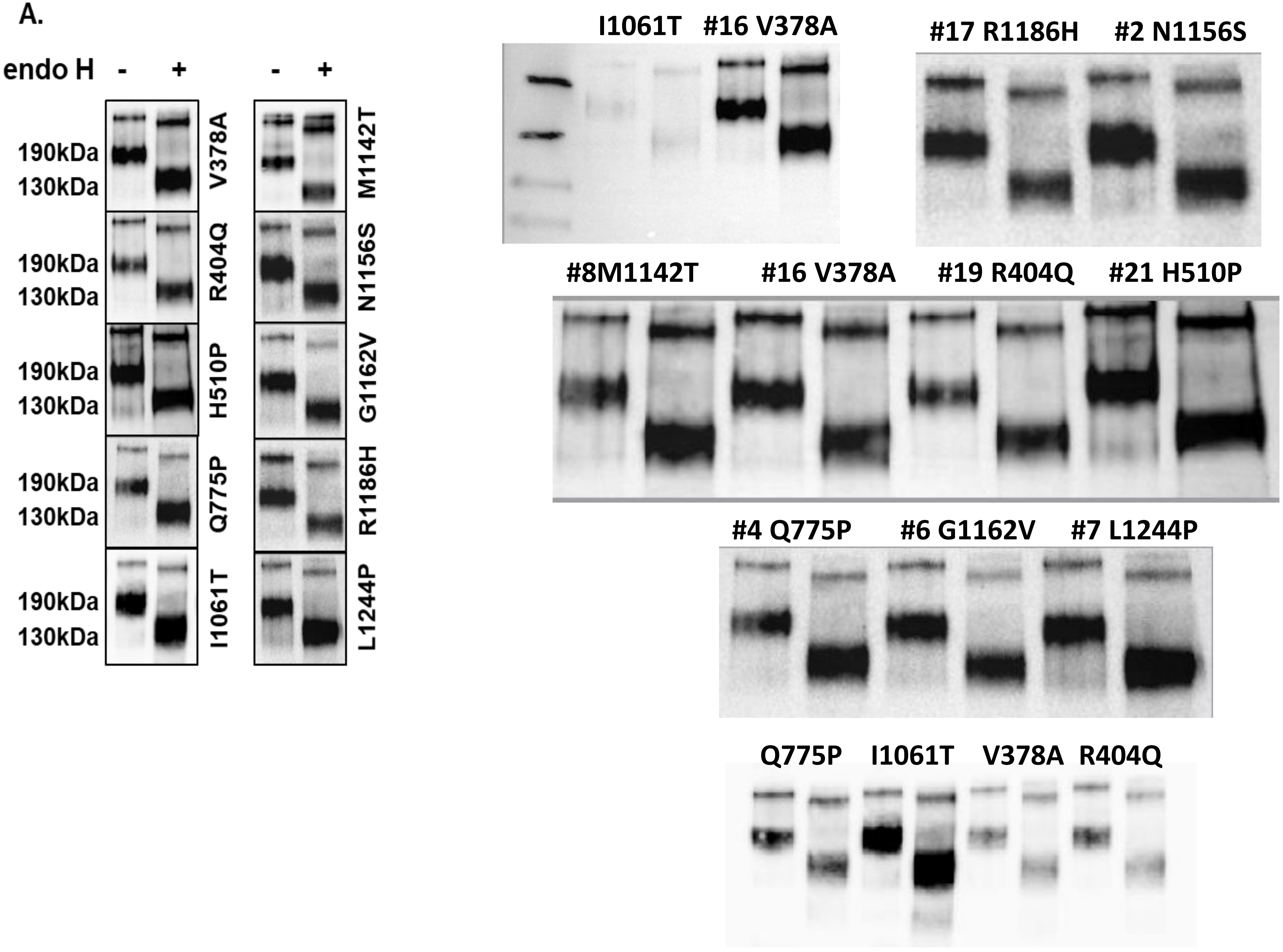

A.
endo H - + - +
190kDa
130kDa
V378A
R404Q
H510P
Q775P
I1061T
M1142T
N1156S
G1162V
R1186H
L1244P
I1061T #16 V378A
#17 R1186H #2 N1156S
#8M1142T #16 V378A #19 R404Q #21 H510P
#4 Q775P #6 G1162V #7 L1244P
Q775P I1061T V378A R404Q

A.

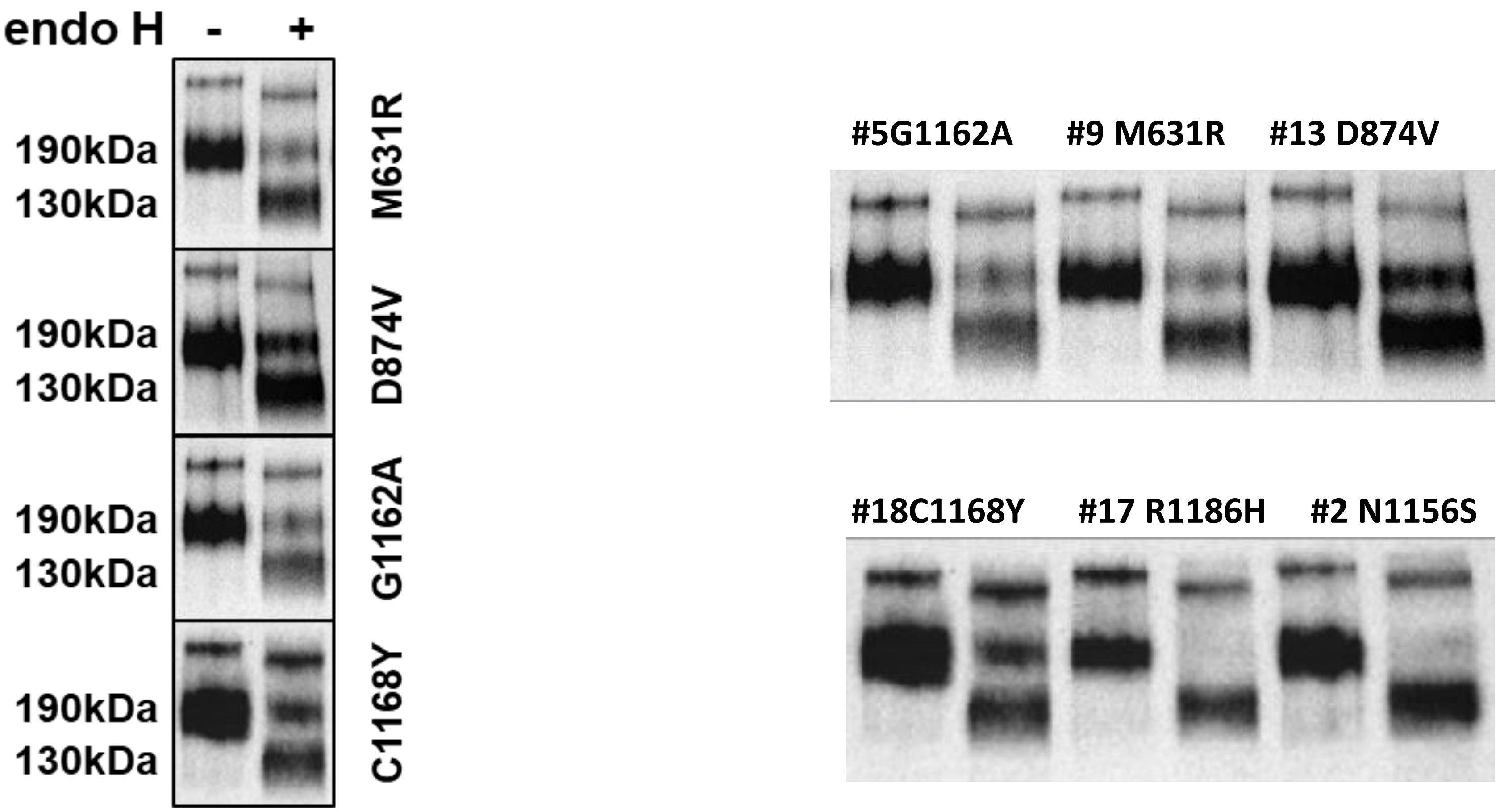
endo H - +
190kDa
130kDa
M631R
190kDa
130kDa
D874V
190kDa
130kDa
G1162A
190kDa
130kDa
C1168Y
#5G1162A
#9 M631R
#13 D874V
#18C1168Y
#17 R1186H
#2 N1156S

A.

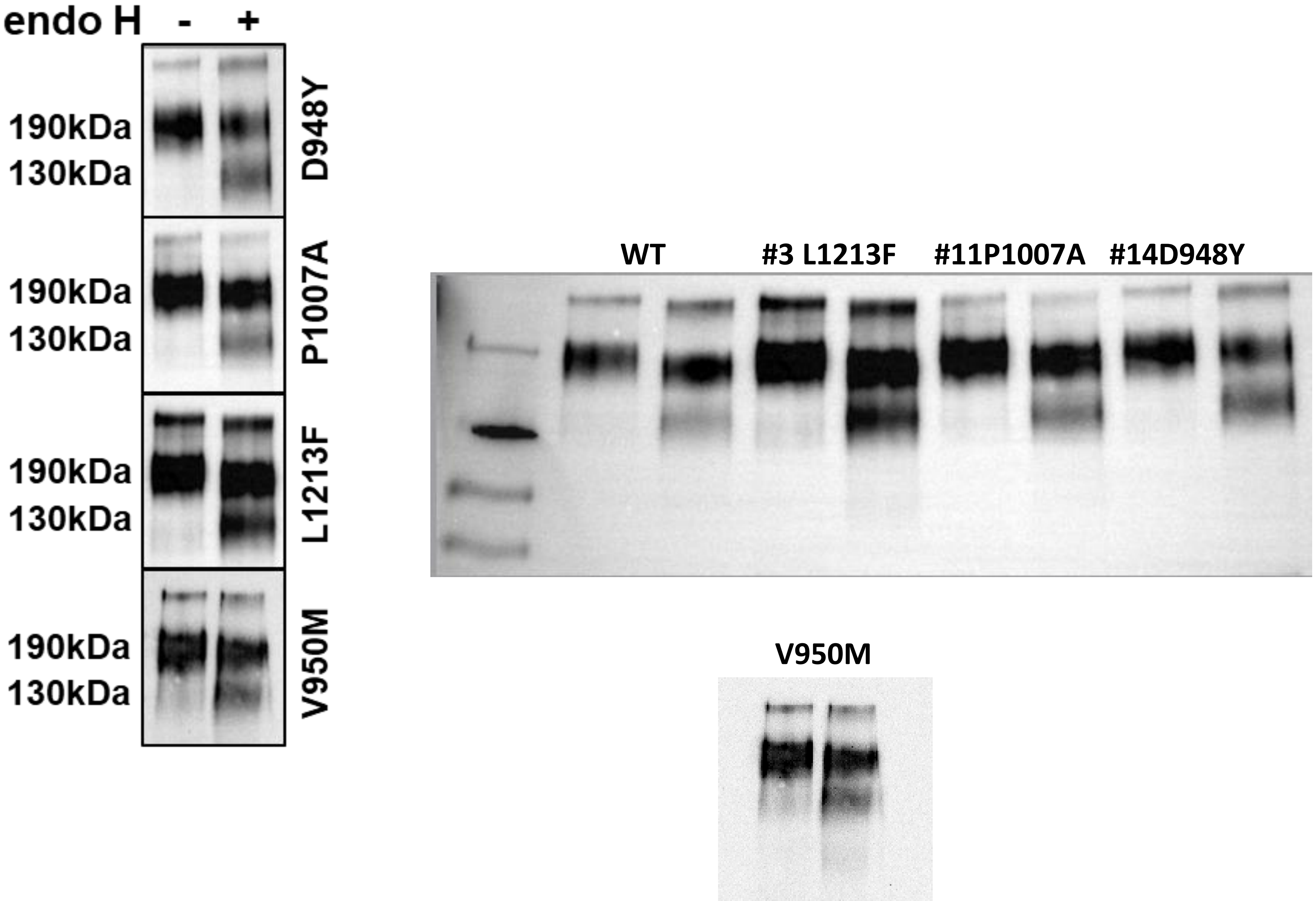
endo H - +
190kDa
130kDa
D948Y
190kDa
130kDa
P1007A
190kDa
130kDa
L1213F
190kDa
130kDa
V950M
WT
#3 L1213F
#11P1007A
#14D948Y
V950M
